# Enzyme-like Enantioselectivity in GTM Chiral Zeolite Catalysts upon Preactivation of Ge Sites

**DOI:** 10.1021/jacs.5c12567

**Published:** 2025-10-08

**Authors:** Ramón de la Serna, Jaime Jurado-Sánchez, Carlos Márquez-Álvarez, M. Asunción Molina, Lucy Costley-Wood, Andrew M. Beale, Diego Gianolio, Joaquín Pérez-Pariente, Luis Gómez-Hortigüela

**Affiliations:** † Instituto de Catálisis y Petroleoquímica, Consejo Superior de Investigaciones Científicas (ICP-CSIC), c/Marie Curie 2, 28049 Madrid, Spain; ‡ Department of Chemistry, 4919 University College London, 20 Gordon Street, London WC1H 0AJ, U.K.; § Research Complex at Harwell, Rutherford Appleton Laboratories, Harwell Science and Innovation Campus, Harwell, Didcot OX11 0FA, U.K.; ∥ 120796 Diamond Light Source, Harwell Science and Innovation Campus, Didcot OX11 0DE, U.K.

## Abstract

Extra-large-pore Ge-containing GTM chiral zeolite catalysts have recently proved useful asymmetric catalysts, with chirality emerging from their chiral confined nanospace. However, so far these exceptional materials have suffered from low framework stability in the presence of water and moderate catalytic enantioselectivity in the ring-opening of chiral *trans*-stilbene oxide with 1-butanol used as a test reaction. Here, we report that these chiral zeolite catalysts can be easily stabilized upon exposure of the calcined material to 1-butanol, providing stability against water and, most importantly, prompting a preactivation of the chiral active sites that boosts their enantioselective properties, reaching unprecedented enantiomeric excesses up to 88% where one enantiomer reacts 16 times more than the other. A range of physicochemical studies, including *in situ* Fourier transform infrared (FTIR) and X-ray absorption spectroscopy, indicates that framework Ge sites increase their coordination environment upon interaction with 1-butanol molecules, which after a thermal treatment above 100 °C remain irreversibly bound to Ge as a consequence of a condensation and dehydration reaction, providing a route to easily functionalize these materials. These preactivated GTM asymmetric catalysts act similarly to enzymes by controlling the confinement of the chiral reactants in particular orientations through coordination with Ge and development of H-bonds with nearby hydroxyl groups, thus attaining enantioselective catalytic activities close to those reached by enzymatic systems but with the crucial advantage associated with heterogeneous catalysts and, notably, the possibility of preparing both enantiomeric versions of the catalyst by using an easily accessible alkaloid.

## Graphical abstract

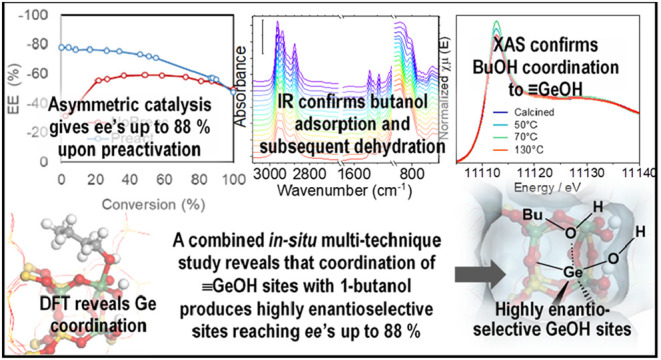

## Introduction

Life is built upon enantiopure chiral entities, l-amino acids and d-sugars, and hence the diastereomeric interactions between organisms and chiral guest species are crucial for chiral recognition mechanisms that are critical for the correct functioning of life. The search for technologies able to enantioselectively prepare chiral compounds represents, nowadays, one of the most formidable challenges in applied chemistry, which is particularly decisive for the pharmaceutical and fine-chemicals industries. Heterogeneous asymmetric catalysis is recognized as the most appealing strategy to attain this goal, but because of the complexity of chiral recognition phenomena, success has been limited. As usual for catalytic systems, evolution allowed nature to provide the best archetypic asymmetric catalysts in enzymes, where the chiral nanospace around the active site emerging from the surrounding enantiopure l-amino acids allows chiral catalytic reactions to occur in an almost enantiospecific manner.^1,2^ Nonetheless, the extraordinary asymmetric activity of enzymatic catalysts is hindered by their homogeneous nature, their low stability under harsh conditions (high temperature or organic solvents), and especially by the limitation imposed by the particular chiral selection of life, which provides exclusively catalysts in one enantiomeric version.^3,4^

When considering the development of more robust chiral heterogeneous catalysts, one naturally turns to inorganic systems, with zeolites representing one of the most powerful catalytic tools.^5–7^ Zeolites are microporous aluminosilicates based on a tetrahedral network with pores of molecular dimensions. Indeed, the chiral confined nanospace found in enzymes can be envisaged as analogous to the chiral nanospace present in chiral zeolite frameworks, while offering the associated advantages of zeolitic materials that include catalytic versatility, hydrothermal stability, and shape-selectivity.^8–15^ Although exhaustively pursued for decades,^16,17^ only recently have chiral zeolites proved successful in promoting enantioselectivity during catalytic reactions. The first example was reported by Davis and co-workers with the STW zeolite framework,^18^ which was recently obtained enantiopure by Sala and co-workers.^19^ However, the application of this material was hampered due to the low enantioselectivity observed (with enantiomeric excess of around 10–20%), the limited scope of asymmetric reactions constrained by its medium pore size (restricting reactions to small chemical entities), the large crystal size, and the low hydrothermal stability of the Ge-containing STW materials. Soon after, we reported the discovery of the GTM series of materials,^20–24^ the first enantio-enriched chiral zeolites based on the -ITV framework,^25^ which represented a milestone in the field of asymmetric catalysis. These GTM materials combined extra-large pores, enabling the processing of bulky molecules, with the chiral nature of their pores that provided asymmetric confined nanospaces where enantioselectivity during catalytic processes emerged, achieving unprecedented enantiomeric excesses up to 60%.^23^ Furthermore, these materials allowed for the preparation of both antipode versions of the catalysts by using very simple chiral precursors derived from ephedrine and pseudoephedrine alkaloids as structure-directing agents (SDAs). However, GTM chiral catalytic materials still suffer from severe drawbacks, particularly their low stability against water due to the presence of Ge, which forces the manipulation of the calcined materials under inert conditions as well as the still limited enantioselectivity achieved in catalytic processes (up to 60%). Here, we report how all these disadvantages can be easily overcome by applying a simple postsynthetic strategy to alter the structure of the chiral active sites, mimicking the behavior of enzymatic asymmetric catalysts.

## Experimental Section

### Synthesis of GTM-3 and GTM-4 Catalysts

(1*R*,2*R*)- and (1*S*,2*S*)-*N*-ethyl-*N*-methyl-pseudoephedrinium (EMPS), (1*R*,2*S*)- and (1*S*,2*R*)-*N*-methyl-*N*-(2-methylbenzyl)-ephedrinium (OMBMEP) and (1*R*,2*R*)- and (1*S*,2*S*)-*N*-methyl-*N*-(2-methylbenzyl)-pseudoephedrinium (OMBMPS) hydroxides, used as structure-directing agents for the synthesis of GTM-3 (EMPS) and GTM-4 (OMBMEP and OMBMPS) materials, respectively, have been prepared from the corresponding commercially available chiral precursors, (1*R*,2*R*)-pseudoephedrine, (1*S*,2*S*)-pseudoephedrine, (1*R*,2*S*)-ephedrine, or (1*S*,2*R*)-ephedrine (Sigma-Aldrich, 98%). Their syntheses, as well as the crystallization of the zeolite materials, have been described already in our previous works.^20,22,23^

### Asymmetric Catalytic Activity of GTM Materials

Calcination of GTM-4 and GTM-3 materials was carried out at 200 °C for 10 h under an ozone-enriched O_2_ stream produced using an Ozonosystem ECO_3_ C-3 electrical discharge ozone generator (ozone concentration *ca*. 50 g/Nm^3^, measured by a BMT 965 ST ozone analyzer); such a low-temperature calcination protocol has been found to minimize the -ITV framework degradation.

The calcined GTM solids were used as acid catalysts for the ring-opening of chiral *trans*-stilbene oxide (TSO) with 1-butanol, a chiral test reaction typically used to characterize the enantioselectivity of chiral zeolite materials.^21^ Catalytic experiments were performed with 20 wt % of catalyst (with respect to *trans*-stilbene oxide), and with 1 mg/mL of racemic *trans*-stilbene oxide solution in 1-butanol (in order to minimize side-reactions); the catalytic reaction was carried out at room temperature (unless otherwise stated) under stirring, and aliquots were extracted at different time intervals. Manipulation of calcined GTM samples was carried out under an inert (N_2_) atmosphere (in a drybox); once butanol has been loaded in the calcined GTM materials, these can then be safely exposed to air. Preactivation treatments involved 1-butanol being loaded in the GTM materials by submerging the calcined materials in 1-butanol, and this suspension was kept under stirring at room temperature overnight, yielding a suspension of the preactivated catalysts in butanol, on which a solution of *trans*-stilbene oxide in 1-butanol was added so that a final total concentration of TSO of 1 mg/mL was obtained. The asymmetric catalytic activity of both nonpreactivated and preactivated GTM catalysts was studied. Evolution of the reaction to give the different products was monitored by HPLC with a chiral stationary phase (Daicel, Chiralcel OD-H, 4.6 mm Ø, 250 mm L, particle size of 5 μm).

### Adsorption and Temperature-Programmed Desorption of 1-Butanol

Adsorption–desorption of 1-butanol on sample GTM-4­(*RS*-OMBMEP) was studied by Fourier transform infrared (FTIR) spectroscopy. The as-made GTM-4­(*RS*-OMBMEP) sample was pressed into a self-supporting wafer (thickness *ca*. 7.0 mg/cm^2^), and the organic structure-directing agent (SDA) was removed by treatment with an ozone-enriched O_2_ stream at 200 °C for 24 h. The detemplated sample wafer was then transferred under dry N_2_ atmosphere to an all-glass transmission cell provided with ZnSe windows, and degassed under dynamic vacuum (residual pressure below 10^–4^ hPa) at 400 °C for 10 h. After cooling down to room temperature, the FTIR spectrum was recorded and showed that all bands corresponding to the organic SDA were fully removed. Then, a series of pulses of 1-butanol vapor (Sigma-Aldrich, ≥99.7%, for HPLC) were introduced in the cell to get a stepwise increase of 1-butanol pressure while keeping the sample wafer at room temperature. A spectrum was recorded after each pulse once a stable pressure was reached, indicating that adsorption equilibrium was attained. Subsequently, 1-butanol pressure was decreased stepwise, and a spectrum was recorded when a stable pressure reading was reached in each step. The sample was finally degassed at high vacuum (<10^–4^ hPa) for 30 min. After the room temperature adsorption–desorption cycle, a temperature-programmed desorption test with FTIR monitoring of the solid (TPD–FTIR) was carried out by heating the sample wafer from room temperature up to 400 °C at a constant rate (4.4 °C/min) under dynamic vacuum and recording periodically the FTIR spectra. All FTIR spectra were acquired in the 4000–650 cm^–1^ wavenumber range, at 4 cm^–1^ resolution, by averaging 128 scans (total collection time 36 s per spectrum), using a Thermo Nicolet Nexus 670 FTIR spectrophotometer equipped with an MCT cryodetector. The corresponding spectrum of the gas phase was subtracted from each spectrum recorded under 1-butanol pressure.

Temperature-programmed desorption with analysis of the evolved gaseous products by mass spectrometry (TPD–MS) was carried out by using a Pfeiffer Vacuum QME 220 quadrupolar mass spectrometer. Around 10 mg of powder sample GTM-4­(*RS*-OMBMEP) as-made was detemplated by treatment with an ozone/oxygen stream at 200 °C for 24 h and transferred to a sealed vial under dry nitrogen in a glovebox. Then, the detemplated sample was put in contact with 1-butanol vapor (Sigma-Aldrich, ≥99.7%, for HPLC) at 50 °C for 30 min. The sample saturated with 1-butanol was introduced in a quartz tube, and this tube was connected to the turbomolecular pump of the spectrometer. The sample was evacuated at room temperature for 1 h, and subsequently, the temperature was increased up to 700 °C at a rate of 5 °C/min. Mass spectra of the gases evolved during heating were acquired in Multiple Ion Detection (MID) measurement mode, in the *m*/*z* range from 10 to 80 amu, with an accumulation time of 200 ms. Mass spectra of reference compounds, namely, water (Milli-Q), 1-butanol (Sigma-Aldrich, ≥99.7%, for HPLC), and 1-butene (Phillips Petroleum, research grade), were also acquired using the same equipment and analysis conditions.

### X-ray Absorption Spectroscopy (XAS) Study of Ge Coordination during 1-Butanol Adsorption on GTM-4

The coordination environment of germanium in GTM-4 during 1-butanol adsorption was studied by *in situ* X-ray Absorption Spectroscopy (XAS). Experiments were performed at the B18 beamline, Diamond Light Source synchrotron (Didcot, U.K.), under proposal SP38597–1. The calcined GTM-4 material was diluted with boron nitride in a 1:50 ratio, pelletized, and sieved to a 250–105 μm fraction. Approximately 50 mg of the sieved sample was loaded into a quartz capillary flow reactor available at beamline B18 and immobilized between two quartz wool plugs. This setup was coupled to a flow system connected to a bubbler containing 1-butanol, enabling controlled adsorption and thermal treatment experiments under defined flow and temperature conditions. XAS measurements were performed at the Ge K-edge (11.1 keV) in fluorescence mode using a Si(111) double-crystal monochromator and Cr-coated mirrors. Energy calibration was conducted by using a Pt metal foil as a reference standard. Two types of XAS spectra were collected by using a constant energy step of 0.3 eV. X-ray Absorption Near Edge Structure (XANES) spectra were acquired over the energy range 11003–11503 eV with an approximate acquisition time of 1 min, while Extended X-ray Absorption Fine Structure (EXAFS) spectra were recorded across the 11003–11953 eV range with a total acquisition time of about 3 min.

The *in situ* experiment proceeded as follows. Initially, the EXAFS spectra of the calcined GTM-4 sample were recorded at room temperature. Subsequently, 1-butanol adsorption was initiated by passing a helium flow (30 mL/min) through a bubbler containing the alcohol at room temperature, saturating the carrier gas before it was directed over the sample. The temperature of the sample was then increased from room temperature to 90 °C at a rate of 5 °C/min. During this heating phase, only XANES spectra were collected to ensure a high temporal resolution. Isothermal holds were implemented at 50, 70, and 90 °C for approximately 20 min each, during which EXAFS data were acquired to monitor changes in the local coordination environment of Ge under adsorption conditions. In the final stage, the sample underwent thermal treatment under continuous helium flow with the temperature ramped from 90 to 200 °C at the same rate. Due to signal deterioration attributed to thermal effects at elevated temperatures, only XANES spectra were recorded during this phase.

Data processing and analysis were conducted using the Demeter software suite.^26^ XANES simulations have been performed using FEFF 10 code,^27^ starting from model structures optimized by DFT as described in the next section. The electronic density was optimized with a Self-Consistent Field (SCF) using a radius of 4 Å including 25 atoms. Full multiple scattering contributions were then calculated using a radius of 6 Å including 53 atoms over an energy range from −10 to 60 eV across the absorption edge.

### Computational DFT Study

A large cluster consisting of 274 atoms (taken from the *P*4_1_32 polymorph) was used as -ITV model (with 73 T atoms), with a composition of Ge_3_Si_70_O_106_(OH)_15_(H_t65_), where interrupted Si positions were saturated with H atoms (H_t_), and with Ge in the *d4r* unit with two interrupted positions in T7, T7 and adjacent T6 positions (this had been previously found as the most stable case).^24^ In order to keep the -ITV framework structure, only atoms in the Ge-containing *d4r* unit and up to its second coordination shell as well as all the terminal OH groups and the organic guest species were allowed to relax during geometry optimizations. Calculations (geometry optimization and transition-state search calculations) were performed with the cluster model at the DFT + D level, with PBEsol functional^28^ and the Tkatchenko and Scheffler dispersion term,^29,30^ using a DNP+ (double numerical plus polarization with diffuse functions) as implemented in DMol3 code. For the calculation of the transition states, an initial guess was estimated by selecting an appropriate interatomic distance as a reaction coordinate and performing constrained geometry optimizations at different fixed intervals. The structure and activation energy of the transition state were then refined by using linear synchronous transit (LST) and quadratic synchronous transit (QST) methods; the structure of the transition state was confirmed by calculating vibrational frequencies. Estimation of the free energies was carried out by adding the entropic contribution at a given temperature (298 K) after calculation of the Hessian; free energies are reported in kcal/mol.

The most stable orientation of TSO enantiomers within the preactivated catalysts was found by a combination of simulated annealing force field-based calculations (using the Dreiding force field)^31^ followed by DFT geometry optimization (in the same conditions as before) of the global minima found previously.

## Results

### Asymmetric Catalytic Activity of 1-Butanol-Preactivated GTM-4

When examining the asymmetric catalytic activity of our GTM chiral catalysts during the ring-opening of *trans*-stilbene oxide (TSO) with 1-butanol, the reaction that we use as a chiral test (see Scheme S1 in the Supporting Information), a surprising behavior was observed: the enantiomeric excess (*ee*) of the main “*unlike*” products that result from the S_N_2 attack of 1-butanol to the oxirane ring (giving (1*R*,2*S*)-2-butoxy-1,2-diphenyl-ethanol (hereafter referred as *RS-unlike*) from (1*R*,2*R*)-TSO, and (1*S*,2*R*)-2-butoxy-1,2-diphenyl-ethanol (*SR-unlike*) from (1*S*,2*S*)-TSO) increased progressively along the course of the reaction up to 30% of conversion (Figure 1). This occurred not only for GTM-4 catalysts prepared from (1*R*,2*S*)-*N*-methyl-*N*-(2-methylbenzyl)­ephedrinium hydroxide (*RS*-OMBMEP) as SDA (Figure 1A, red line), with the *ee* increasing from −30% at the beginning up to −59% at midconversion, but also for those prepared from (1*S*,2*S*)-N-ethyl-*N*-methyl-pseudoephedrinium hydroxide (*SS*-EMPS) as SDA (Figure S1 in the Supporting Information-top, red line), increasing from −12 to −48%, and also for GTM-4 prepared from (1*S*,2*S*)-*N*-methyl-N-(2-methylbenzyl)-pseudoephedrinium hydroxide (*SS*-OMBMPS) (Figure S2 in the Supporting Information), which increased from +35 to +55% (in the latter case the opposite sign involves an antipode chirality of the -ITV framework).^22^ This surprising behavior suggested that the enantioselective active sites underwent some kind of transformation during the course of the reaction that strongly activated their enantioselective properties. This behavior resembled that of some enzymes, which in some cases require the action of cofactors that induce some local modifications on the active sites that partially alter the chiral environment and trigger their enantioselectivity. In fact, such modifications of the active site of enzymes can be accomplished by solvent molecules themselves that associate with the chiral nanospace environment and induce a stronger enantiomeric recognition ability.^32,33^ Given such unexpected behavior, we opted to follow a similar strategy and tactically preactivate our GTM catalysts in a way that mimics the reaction conditions, which could be through interaction with either reactant, TSO or 1-butanol. Preactivation with TSO was not possible since, in the absence of a nucleophile, TSO is rapidly transformed into diphenylacetaldehyde by our GTM catalysts. Hence, we opted to preactivate the catalysts by treating them with 1-butanol prior to the reaction (by submerging in neat 1-butanol under agitation at room temperature overnight) and then performing the catalytic process under the same conditions as before. Interestingly, such preactivation treatment boosted a prominent improvement of the enantioselectivity of the catalyst (Figure 1), where the *ee* of the main “*unlike*” products (Figure 1A) increased from −30 to up to −78% (at low conversion, or from −59 to ∼−75% at midconversion) for GTM-4­(*RS*) catalyst (note that the *ee* decreased after midconversions due to the disappearance of the most reactive TSO reactant enantiomer, as typical in this kind of kinetic resolution-like reactions that involve the transformation of racemic chiral reactants). Similarly, the *ee* of reactants (Figure 1B), which increased along the course of the reaction, was also notably enhanced upon preactivation, even reaching 100% at high (80%) conversions, as well as that of the minor “*like*” products that also increased along the reaction (Figure 1C). Such preactivation treatment prompted a slight enhancement of the selectivity to the main “*unlike*” products with a corresponding reduction of the “*like*” products (Figure S3 in the Supporting Information). Together with the increase in both *ee*’s (“*unlike*” and “*like*” products), these results suggest an enhanced preference for the main S_N_2 reaction pathway for the most reactive TSO enantiomer and a preferred S_N_1 path for the less reactive TSO enantiomer upon preactivation. As expected, a reaction performed with antipode GTM-4 catalysts (prepared from (1*R*,2*S*)- or (1*S*,2*R*)-enantiomers of the OMBMEP SDA) gave exactly the same behavior with opposite signs (Figure S4 in the Supporting Information). A similar behavior is observed for GTM-3 catalyst (prepared from *SS*-EMPS), where preactivation also induced an improvement of the enantioselectivity of the reaction (with *ee’*s for the “*unlike*” products enhanced from −27 to −64% at low conversion) (Figure S1 in the Supporting Information), and similarly for GTM-4 prepared from *SS*-OMBMPS (with *ee*’s for the “*unlike*” products rising from +35 to +71% at low conversion) (Figure S2 in the Supporting Information), indicating that the effect of preactivation on the enantioselectivity is inherent to the Ge-containing -ITV framework regardless of the SDA used in the synthesis. In any case, it is worth noting that the highest enantioselectivity reached after preactivation was achieved by the GTM-4 catalyst prepared from *RS*-OMBMEP (Figure S5 in the Supporting Information), up to 78% under these conditions.

**Figure 1:**
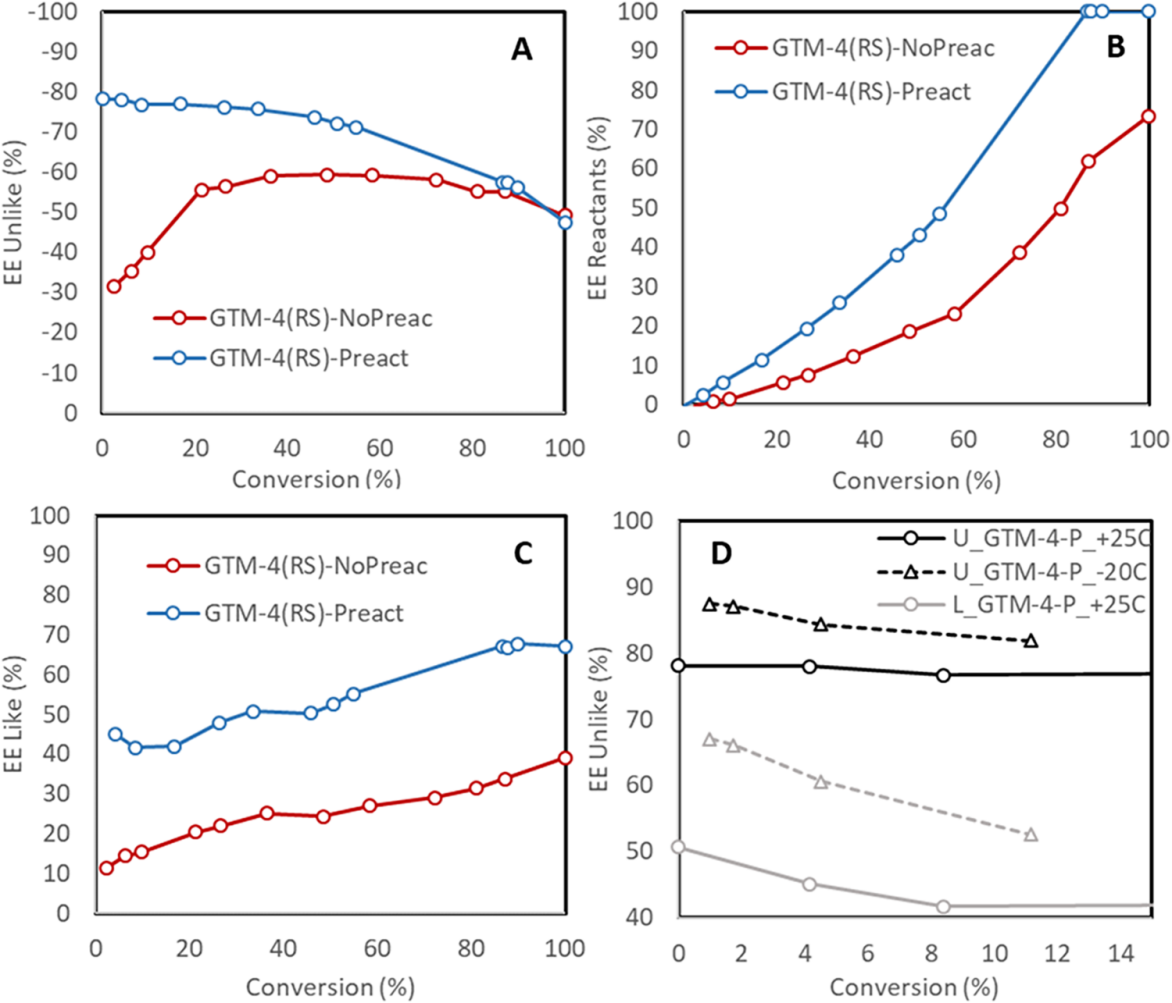
Asymmetric catalytic activity (reported as enantiomeric excess of reactants (B), “*unlike*” products (A), and “*like*” products (C)), of GTM-4 (prepared from (1*R*,2*S*)-*N*-methyl-*N*-(2-methylbenzyl)­ephedrinium hydroxide) as-calcined (red lines) and preactivated (blue lines) catalysts for the *trans*-stilbene oxide ring-opening with 1-butanol at room temperature. (D): Effect of reaction temperature with preactivated GTM-4 catalyst (note that for comparison purposes, in this case we report the absolute values of the *ee* for each product, keeping the same sign: *ee* of “*unlike*” products have been multiplied by −1).

In an attempt to further improve the enantioselectivity of our GTM-4 catalyst, and given the fact that the *ee* of the “*unlike*” products seems to be associated with a higher selectivity to these S_N_2 products (and a decrease to the “*like*” S_N_1 products, see Figure S3 in the Supporting Information), we lowered the reaction temperature from 25 to −20 °C (Figure 1D), which made the reaction rate much slower and more selective. Interestingly, an outstanding *ee* of up to −88% (at the beginning of the reaction, black dashed line) was reached (compared to −78% at room temperature, solid black line); such enantioselectivity has no precedent in the field. Similarly, the *ee* to the “*like*” products also increased from 50 to 67% (gray lines).

### Stability of Preactivated GTM-4 Samples

Given the excellent performance of such preactivated catalysts (in particular, for GTM-4 prepared from *RS*-OMBMEP, Figure S5), we studied the effect of the preactivation treatment over these GTM-4 materials. First, we analyzed the effect on the stability of the calcined GTM-4 materials (Figure 2). XRD results showed that the crystalline structure of GTM-4 was preserved after removal of the organic SDA by ozone treatment at moderate-temperature (200 °C) when this calcined sample was manipulated under a dry inert atmosphere (Figure 2A, blue line, calcined sample protected with Kapton film), but as soon as it was exposed to ambient air, the framework immediately collapsed due to the effects of humidity (red line), evidencing the necessity of manipulation of the calcined samples under an inert atmosphere. In contrast, if the materials are subjected to the preactivation treatment to fill the pores with butanol (at 50 °C, see Figure S6 in the Supporting Information, where the incorporation of butanol is evidenced by thermogravimetric analysis), they become completely stable against exposure to ambient air even after 168 h (Figure 2B). This clearly demonstrates that the incorporation of butanol into the pores of the -ITV framework results in a convenient stabilization of these materials against hydrolysis, which is crucial for their potential catalytic application. Repetition of these stability studies under different preactivation conditions, specifically after submersion in butanol at 115 °C, showed similar results (Figure S7 in the Supporting Information).

**Figure 2:**
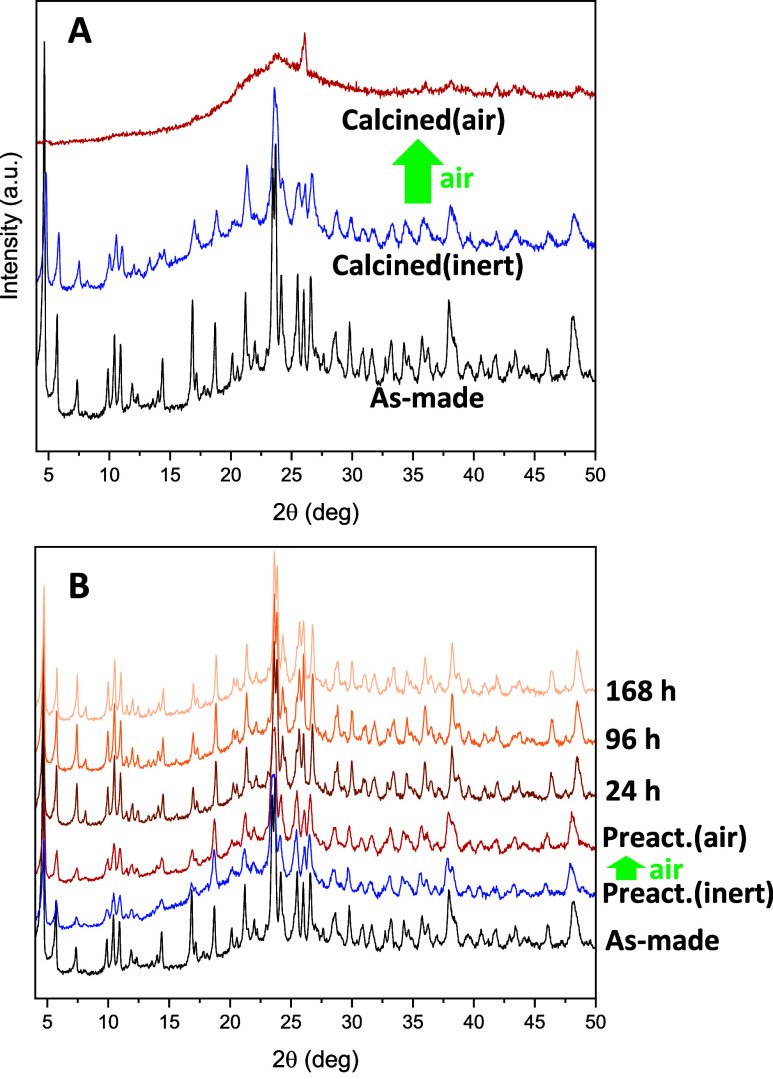
XRD patterns of GTM-4­(*RS*-OMBMEP) untreated (A, top) or after preactivation in 1-butanol at 50 °C (B, bottom). From bottom to top: as-made, calcined (XRD analysis performed under inert atmosphere), calcined (immediately after exposure to ambient air), and preactivated sample after 24, 96, and 168 h under air. Note: the higher baseline in the calcined-inert samples (blue lines) is caused by the Kapton film that maintains the dry conditions.

### *In Situ* FTIR Study of 1-Butanol Adsorption in Gas Phase

Given the thrilling effect of the incorporation of 1-butanol within the -ITV pores on the asymmetric catalytic activity as well as on the framework stability, we analyzed the gas phase adsorption of butanol on calcined GTM-4 by *in situ* FTIR (Figure 3). Calcined GTM-4 displays two sharp bands at 3747 and 3679 cm^–1^ that correspond to OH stretching of ≡Si–OH and ≡Ge–OH free groups, respectively.^34–37^ Upon adsorption of butanol at increasing equilibrium pressures (Figure 3A), the band assigned to ≡Ge–OH free groups continuously decreases while bands corresponding to 1-butanol start to appear (the most prominent being the C–H stretching and deformation bands in the 2800–3000 and 1350–1500 cm^–1^ wavenumber ranges, respectively); however, the band corresponding to ≡Si–OH is not altered until reaching a butanol pressure of 16 Pa, when it starts to progressively decrease. At the same time, the band at 772 cm^–1^, which we assign tentatively to the bending of ≡Ge–OH groups, also disappears. These results clearly indicate a strong preference of adsorbed butanol molecules to interact with ≡Ge–OH free groups. Concomitant to the depletion of ≡Ge–OH and ≡Si–OH hydroxyl stretching bands, a broad absorption in the range 3100–3600 cm^–1^ develops. This absorption appears to be the envelope of several broad overlapping bands, which can be attributed to hydroxyl stretching of adsorbed butanol as well as surface ≡Ge–OH and ≡Si–OH groups strongly distorted due to hydrogen bonding. Subsequent decrease of butanol equilibrium pressure (Figure 3B) leads to a minor decrease of butanol C–H stretching and bending bands, even under a high vacuum, indicating the release of only a small fraction of the adsorbed butanol. Also, the broad absorption in the hydroxyl stretching region decreases, mainly in the 3200–3400 cm^–1^ range, revealing two broad bands with maxima at 3565 cm^–1^, which might be assigned to hydrogen-bonded ≡Ge–OH hydroxyls, and at 3204 cm^–1^, which we assign to OH stretching of adsorbed butanol. The release of butanol under a high-vacuum treatment at room temperature (Figure 3B, red line) leads to a partial recovery of free ≡Si–OH groups (band at 3739 cm^–1^). In contrast, both the hydroxyl deformation band at 772 cm^–1^ and the stretching band at 3679 cm^–1^ of free ≡Ge–OH groups remain fully depleted. From these results, we can conclude that butanol molecules interact preferentially with ≡Ge–OH sites and that they remain adsorbed on those sites at room temperature after the high-vacuum treatment.

**Figure 3:**
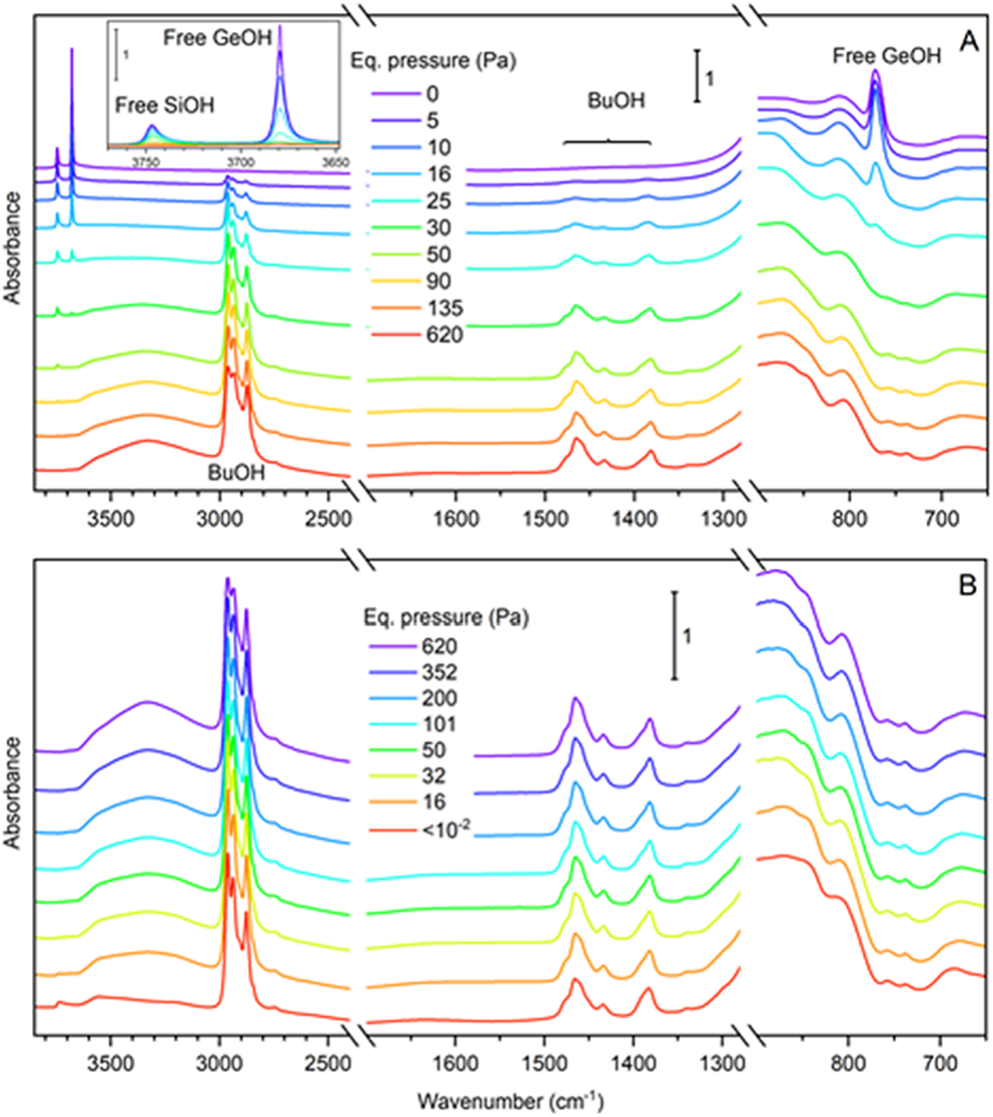
Adsorption of 1-butanol at room temperature on the sample GTM-4­(RS-OMBMEP). (A) FTIR spectra recorded after reaching progressively increasing equilibrium pressure of 1-butanol vapor. Inset shows an enlarged view of the OH stretching bands region of ≡Si–OH and ≡Ge–OH groups. (B) FTIR spectra recorded during 1-butanol desorption after reaching progressively decreasing equilibrium pressure of 1-butanol. The last spectrum (red line) was recorded after 30 min at the highest attainable vacuum.

After the room temperature 1-butanol adsorption–desorption test carried out in the FTIR cell, the sample was heated at a constant rate under a vacuum to perform a temperature-programmed desorption test with FTIR monitoring of the solid (TPD–FTIR). The series of spectra recorded during heating (Figure 4) indicated a progressive, partial loss of butanol with increasing temperature. However, even at 397 °C under a vacuum, the C–H stretching and bending bands of adsorbed butanol remain intense, showing only some thermal broadening. These results indicate that most of the butanol molecules remained adsorbed on GTM-4 even at high temperatures, revealing a very stable bond with ≡Ge–OH sites. Indeed, the spectrum of the sample recorded at the highest temperature shows nearly 60% of the integrated band area in the region of methyl and methylene stretching modes (3055–2785 cm^–1^) compared to the initial spectrum recorded at room temperature. Interestingly, the band at 3565 cm^–1^, assigned to the O–H stretching of ≡Ge–OH sites interacting with adsorbed 1-butanol molecules, first shifts toward higher wavenumbers up to 3579 cm^–1^ at 89 °C, after which it completely vanishes at temperatures beyond 115 °C. Simultaneously, the band at 3204 cm^–1^ assigned to O–H stretching of adsorbed butanol decreases quickly with temperature up to 115 °C and vanishes below ca. 300 °C. Moreover, the band at 772 cm^–1^ assigned to O–H deformation of ≡Ge–OH sites is not recovered. The disappearance of both O–H bands (of ≡Ge–OH and BuOH species) suggests a dehydration process to produce butoxide species at temperatures around 115 °C that remain bound to Ge sites up to, at least, *ca*. 400 °C. This transformation of adsorbed butanol into butoxide species agrees with the transformation from H-bonding to butanol coordination and then butoxide formation upon Ge sites as evidenced by XANES analysis (see below) and might also explain the change of CH stretching bands of the alkyl chain of butanol observed above 115 °C. This region of the spectra remains unaltered below 115 °C, except for an overall progressive decrease of intensity. However, at higher temperatures, these bands show a slight shift and a change of their relative intensities. Thus, the intensity of the asymmetric stretching band of methyl (ca. 2960 cm^–1^) relative to methylene groups (ca. 2940 cm^–1^) remains constant from room temperature to 115 °C, and it shows a progressive decrease from this temperature until it reaches a constant value above 370 °C (Figure S8 in the Supporting Information). Similar changes can also be observed in the methylene and methyl deformation bands (1350–1470 cm^–1^).

**Figure 4:**
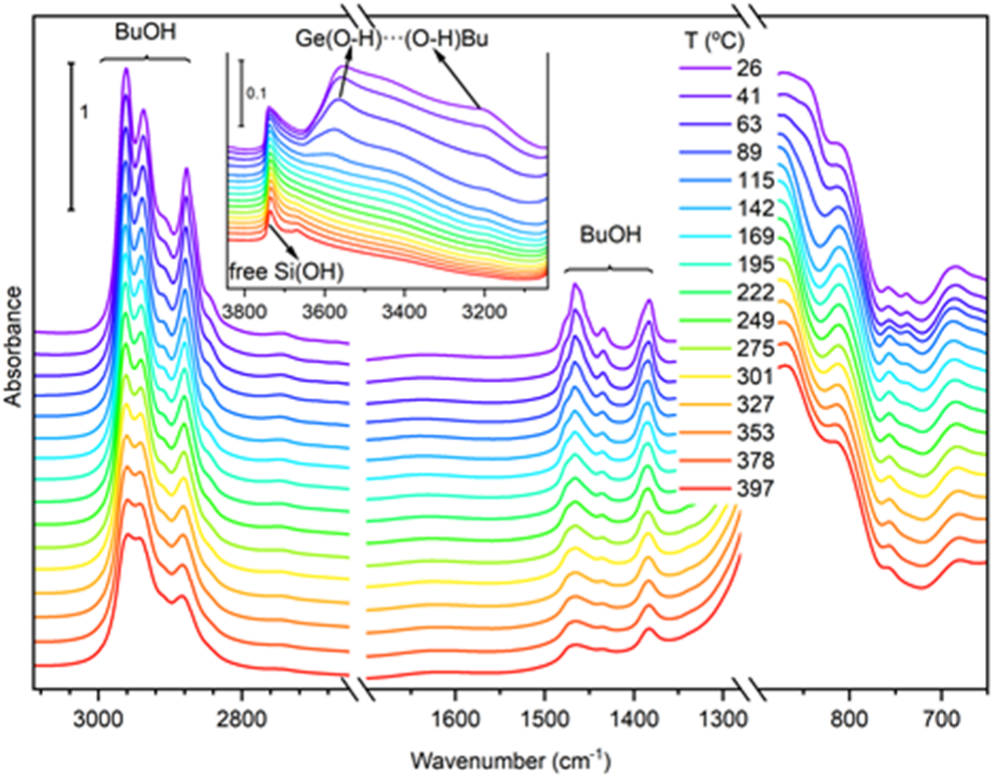
TPD–FTIR analysis of sample GTM-4­(*RS*-OMBMEP) with 1-butanol preadsorbed at room temperature. The inset shows an enlarged view of the OH stretching region.

^13^C CP MAS NMR (Figure S9 in the Supporting Information) demonstrated the incorporation of 1-butanol within the pores, where all four resonances of butanol were observed. Two signals for the methylene C atoms bonded to O were observed at 64 and 66 ppm when the preactivation treatment was performed at 50 °C, possibly associated with two environments of butanol molecules, one bound to ≡Ge–OH sites, and another one confined by interaction with the framework walls, while only one was observed at 115 °C, assigned to the presence of butanol/butoxide species bound to Ge sites. CHN elemental analysis gave a butanol content (in the material where butanol was loaded at 50 °C) of around 34 molecules (9.28% C, 2.51% H) per -ITV unit cell (that contains 32 ≡Ge–OH sites), resulting in a nearly 1:1 ratio of 1-butanol to ≡Ge–OH (1.06).

### TPD–MS Analysis of Preactivated GTM-4

In order to get deeper insights on the interaction of 1-butanol with GTM-4 zeolite, we analyzed by mass spectrometry the gases evolved during a temperature-programmed desorption (TPD–MS analysis) of sample GTM-4­(*RS*-OMBMEP) with preadsorbed 1-butanol. The spectra showed, as expected, the formation of ions with a *m*/*z* ratio of 31, the main fragment in the 1-butanol MS pattern (Figure 5). This is the most intense signal obtained at temperatures around 100 °C, and its evolution is paralleled by less intense signals at *m*/*z* values corresponding to other characteristic fragments of 1-butanol (such as 27, 28, 29, 41, 42, 43, and 56 amu, among other minor fragments). These data prove that a 1-butanol desorption event occurs at temperatures around 100 °C. Furthermore, strong signals at *m*/*z* ratios 17 and 18 suggest that water desorption also takes place simultaneously.

**Figure 5:**
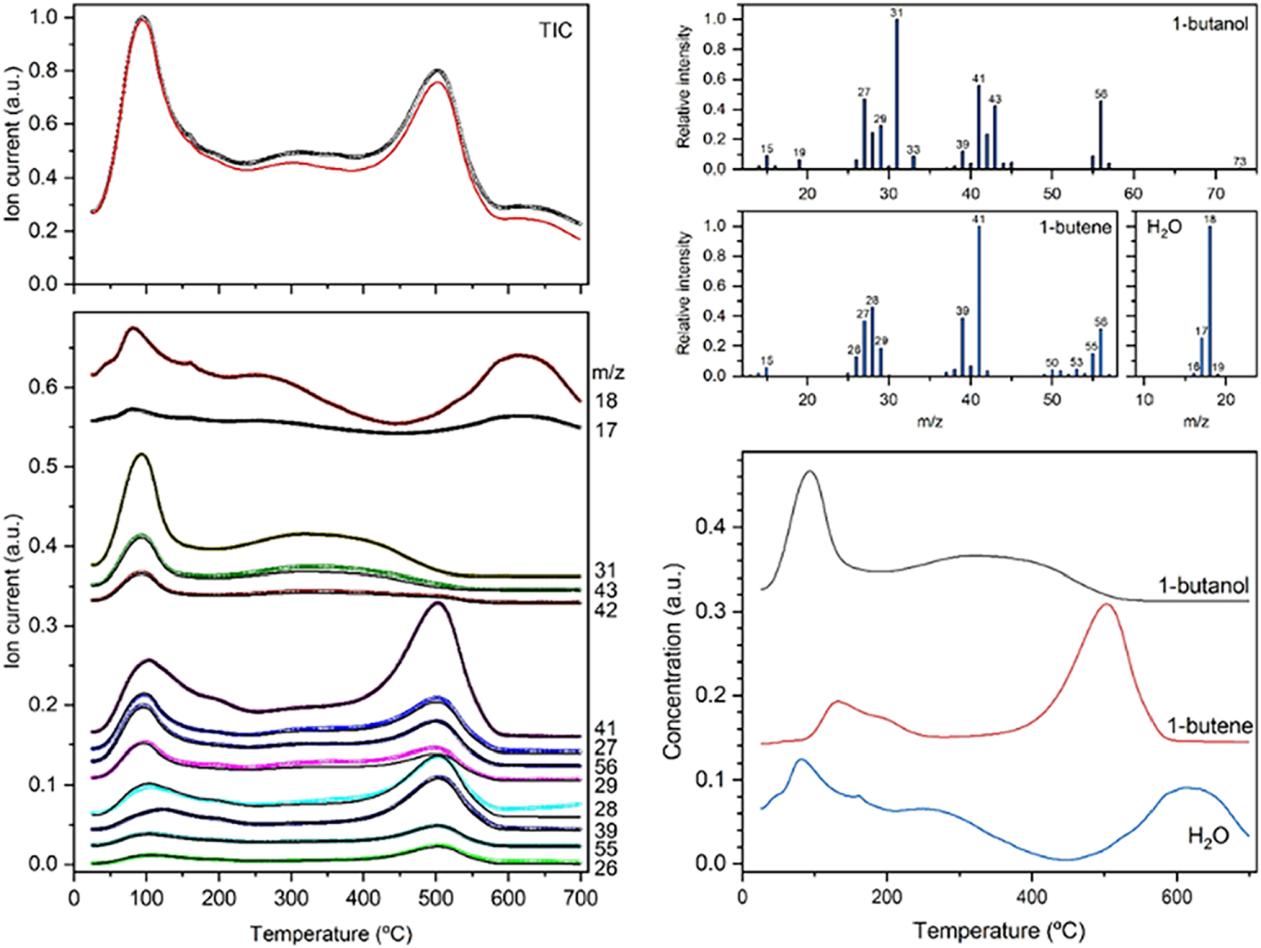
TPD–MS analysis of sample GTM-4­(*RS*-OMBMEP) with preadsorbed 1-butanol. Left: total ion current (top) and ion current intensity for ions with selected (most intense) *m*/*z* values (bottom). Circles correspond to measured ion current, and lines to fitted values. Right: Measured mass spectra of reference compounds used for fitting (top) and calculated concentration profiles (shifted vertically for clarity) of the identified products that desorb as a function of the temperature (bottom).

At temperatures around 500 °C, the spectra show an intense signal corresponding to ions with an *m*/*z* ratio of 41 (the second most intense signal in the MS pattern of 1-butanol), as well as peaks with a similar profile corresponding to several ions found in the MS pattern of 1-butanol. However, those signals are not accompanied by a corresponding increase of intensity at *m*/*z* 31 (the main fragment of 1-butanol), which indicates that a product other than 1-butanol desorbs at a high temperature. Taking into account the similarities in the MS spectra of this additional species and that of 1-butanol, as well as the lack of fragments with *m*/*z* ratio of 31 (which corresponds to the CH_2_OH^+^ ion formed by cleavage of the bond between α and β carbons in 1-butanol), we considered 1-butene as a potential candidate for the compound released at high temperature. Therefore, we collected the MS spectra for the three potential desorption products identified (see patterns in Figure 5, top right) and fitted the whole series of MS spectra recorded along the TPD to a linear combination of the patterns of the three pure compounds. Figure 5 shows that a combination of the patterns of the three reference compounds (water, 1-butanol, and 1-butene) fits well most of the experimental total ion current (TIC) at all temperatures and closely matches the profiles of the most intense signals measured. These results strongly support the hypothesis that water, 1-butanol, and 1-butene are virtually the only desorption products.

The calculated desorption profiles for water, 1-butanol, and 1-butene are plotted in Figure 5 (bottom, right). A first desorption peak of 1-butanol takes place in the 40–140 °C temperature range, with a maximum at 95 °C, in agreement with the TPD–FTIR results discussed above. Also, a peak assigned to 1-butene is detected at temperatures from 100 to 230 °C. This can be attributed to the dehydration of 1-butanol, which might be catalyzed by the zeolite acid sites. A water desorption peak observed at slightly lower temperatures might be attributed to the removal of adsorbed water. However, according to the sample preparation procedure, we believe that the amount of adsorbed water should be negligible. This hypothesis is also supported by the lack of a band due to a water bending mode at around 1640 cm^–1^ in the FTIR spectra of the sample submitted to 1-butanol adsorption at room temperature (Figure 3). Therefore, we assign this first water desorption peak (at least partially) to the formation of butoxide species by the condensation of adsorbed 1-butanol and a neighboring hydroxyl group. Partial dehydroxylation seems to occur also at temperatures above 200 °C as indicated by the formation of water. The presence of water at those temperatures might promote hydrolysis of some of the butoxide species formed at lower temperature, which could explain the second and very broad desorption peak of 1-butanol that extends from ca. 200 to 500 °C. However, most of the butoxide species, which according to the TPD–FTIR data remain linked to the zeolite surface up to at least 400 °C, seem to decompose at slightly higher temperatures releasing 1-butene, which shows a strong desorption peak with a maximum at *ca*. 500 °C (Figure 5). At 600 °C, butanol and butoxide species would be fully removed, as no further release of organic compounds is observed. At this high temperature, only the release of water is observed, probably corresponding to complete dehydroxylation and collapse of the zeolite structure.

### *In Situ* EXAFS/XANES Study of Ge Environment upon 1-Butanol Preactivation

To investigate how the local environment of Ge sites is affected by the adsorption of 1-butanol, an *in situ* EXAFS/XANES experiment was performed under a flow of 1-butanol at different temperatures (Figure 6). It is important to note that not all Ge atoms are located at the ≡Ge–OH Q^3^ sites, where adsorption is expected to occur. Given the high Ge content, some Ge atoms would be located at Q^4^ sites, embedded within double four-ring (*d4r*) units, which in principle should remain unaffected by butanol (as previously mentioned, CHN analyses indicate a ratio of 1-butanol to ≡Ge–OH close to 1). Therefore, considering that XAS provides an average signal over all Ge sites, our analysis focuses on differential changes rather than attempting a fully quantitative evaluation.

**Figure 6:**
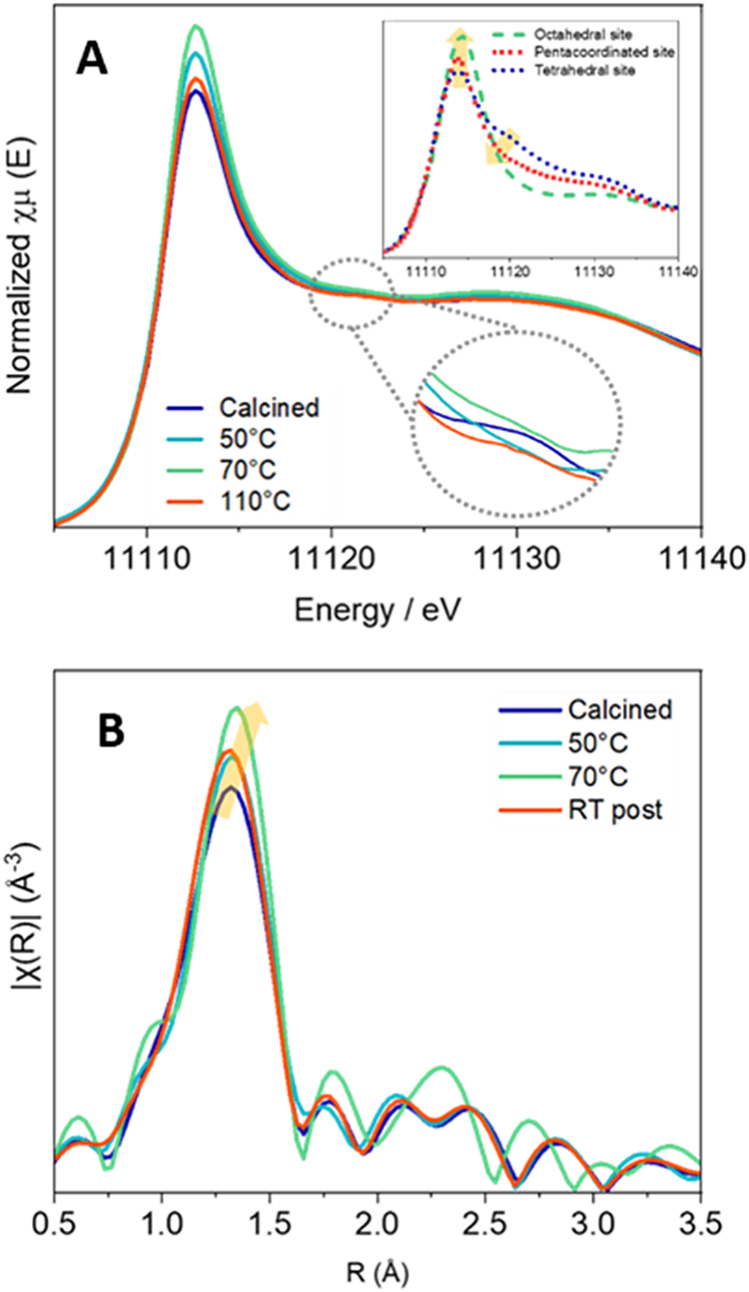
During the *in situ* XAS experiment, calcined GTM-4 (dark blue) was exposed to a flow of 1-butanol and subsequently heated at different temperatures ranging from room temperature to 200 °C. Left: Normalized XANES spectra at the Ge K-edge for GTM-4 were obtained throughout the *in situ* experiment. For comparison, simulations performed with FEFF 10 (0.2 eV resolution) for isolated tetrahedral, pentacoordinated, and octahedral Ge sites in the model generated using DFT are included as references (inset). Additionally, an enlargement of the post-edge region is provided for better visualization of the results. Right: Fourier transform (FT) magnitudes weighted by *k* ^2^ for GTM-4 during the *in situ* experiment (*k*-range of 2.7–14.2 Å^–1^).

The sequence of XANES spectra collected at different reaction conditions (Figure 6A) shows a clear trend: starting from the calcined sample (dark blue line), the adsorption of 1-butanol (after passing the flow) did not alter too much the intensity of the white line (only a slight increase is appreciated; see Figure S10 in the Supporting Information), suggesting that 1-butanol is mostly initially adsorbed through H-bonds, in line with the FTIR observations. Then, as the temperature increases from room temperature (RT) to 70 °C (green line), there is a progressive increase in both the intensity and width of the white line, accompanied by the disappearance of a post-edge feature at approximately 11122 eV (see inset in Figure 6A). At 110 °C (red line), this trend reverses and the spectrum becomes more similar to that of the calcined sample (Figure 6A). To help interpret the spectral differences noticed experimentally and illustrate the expected changes as a function of the Ge local environment, three XANES spectra were simulated (see the inset of Figure 6A). The simulated spectra are based on structural models representing distinct coordination environments: a tetrahedral geometry for the calcined sample, and a pentacoordinated and an octahedral geometry assuming the coordination of one or two butanol molecules to a single Ge site, respectively. The simulations indicate that the lower white line intensity and the presence of a post-edge feature are characteristic of tetrahedral coordination, while an increase in coordination number (pentacoordinated or octahedral geometry) results in a stronger white line and the disappearance of the post-edge feature. These results support the interpretation that the observed spectral changes reflect a structural transformation from a fully tetrahedral environment in the calcined sample to a more centrosymmetric geometry (pentacoordinated or octahedral) upon heating at 70 °C under a 1-butanol flow, due to adsorption at reactive Ge sites. The evolution of the white line intensity with temperature (Figure S10 in the Supporting Information, left) reveals that coordination with butanol begins at RT (though to a low extent), increases progressively up to 70 °C, and stabilizes thereafter. No significant spectral changes are observed between 70 and 90 °C. However, further heating from 90 to 110 °C leads to a decrease in the white line intensity and the reappearance of the post-edge feature (Figures 6 and S10 in the Supporting Information, right), indicating a partial recovery of the tetrahedral coordination. This is attributed to the dehydration process and the formation of butoxide species with the release of water with the subsequent decrease of the coordination number, as previously discussed and consistent with the TPD–FTIR/MS results. Additional heating from 110 to 200 °C does not result in further spectral changes, confirming the thermal stability of the species formed.

The EXAFS region (Figure 6B) also exhibits consistent trends among the calcined sample, the samples treated at moderate temperatures (50 and 70 °C, light blue and green lines, respectively), and the sample measured at room temperature after heating to 200 °C (RT post, red line). These specific data sets were selected due to the inherent difficulty of acquiring high-quality EXAFS data over an extended energy range under *in situ* conditions, particularly due to the presence of vapor flow and associated instabilities, and because increased thermal disorder at elevated temperatures can significantly affect the EXAFS signal. The observed EXAFS variations confirm that the local coordination environment of Ge changes during the 1-butanol adsorption process. Nevertheless, due to the coexistence of distinct Ge sites within the zeolite framework and the intrinsic averaging nature of EXAFS, it is not feasible to perform a reliable quantitative fitting to determine the exact coordination numbers. This limitation arises from the simultaneous contribution of both the responsive (Q^3^) and unresponsive (Q^4^) Ge sites. Despite these constraints, valuable structural insights can still be extracted. Notably, an increase in the amplitude of the first-shell signal upon heating up to 70 °C is consistent with an increase in the average Ge coordination number, attributed to 1-butanol adsorption on a fraction of Ge sites. This is followed by a decrease in amplitude upon returning to room temperature after heating to 200 °C, indicating partial restoration of the initial coordination state, likely associated with butanol dehydration and subsequent butoxide formation. At 70 °C, a noticeable shift of the Ge–O peak toward longer distances is observed, which is consistent with a distortion of the local Ge environment due to adsorption. This elongation aligns with previous reports comparing Ge–O bond lengths in octahedral *versus* tetrahedral coordination, such as in rutile-type and quartz-type GeO_2_.^38^ Furthermore, changes in the second-shell region suggest that adsorption induces a partial loss of medium-range order, particularly in the Ge–Ge/Ge–Si scattering paths. Upon the reestablishment of tetrahedral coordination, this order is expected to be at least partially restored. However, the interpretation of the second-shell region remains more complex due to the overlap of multiple scattering contributions and the presence of structural disorder.

### Computational DFT Study

We then performed a computational study based on DFT + D in order to unravel the nature of the bonding of butanol to ≡Ge–OH sites. Two modes of adsorption of butanol on the -ITV framework are possible: (i) through H-bonds with free ≡Ge–OH sites and (ii) through binding with Ge. Adsorption through H-bonds (see two examples in Figure S11 in the Supporting Information) is slightly more exothermic, giving adsorption free energies (at 298 K) of −28.3 kcal/mol (Figure 7B); calculations showed that at least 35% of this interaction occurs *via* intermolecular interactions of the alkyl chain with the framework walls. Due to the Lewis acidity of tetrahedral Ge atoms, they can increase their coordination environment by binding new ligands, as demonstrated by the EXAFS/XANES experiment. Formation of this Ge···(OH)­Bu complex by direct Ge–O­(BuOH) coordinative binding is much more favored for Ge in the T7 position (≡Ge­(T7)–OH sites, Figure 7C), giving an adsorption free energy of −21.6 kcal/mol. The formation of this complex with Ge­(T7) might be favored because of the flexibility of the ≡Ge­(T7)–OH interrupted position and also because the rearrangement of tetrahedral Ge to accommodate an additional ligand (becoming a trigonal bipyramid) involves a distortion of the Ge environment (Figure 7B→C, dashed green arrows) that enables the development of an intraframework H-bond with an adjacent ≡Ge­(T7)­OH (Figure 7C, dotted blue line).

**Figure 7:**
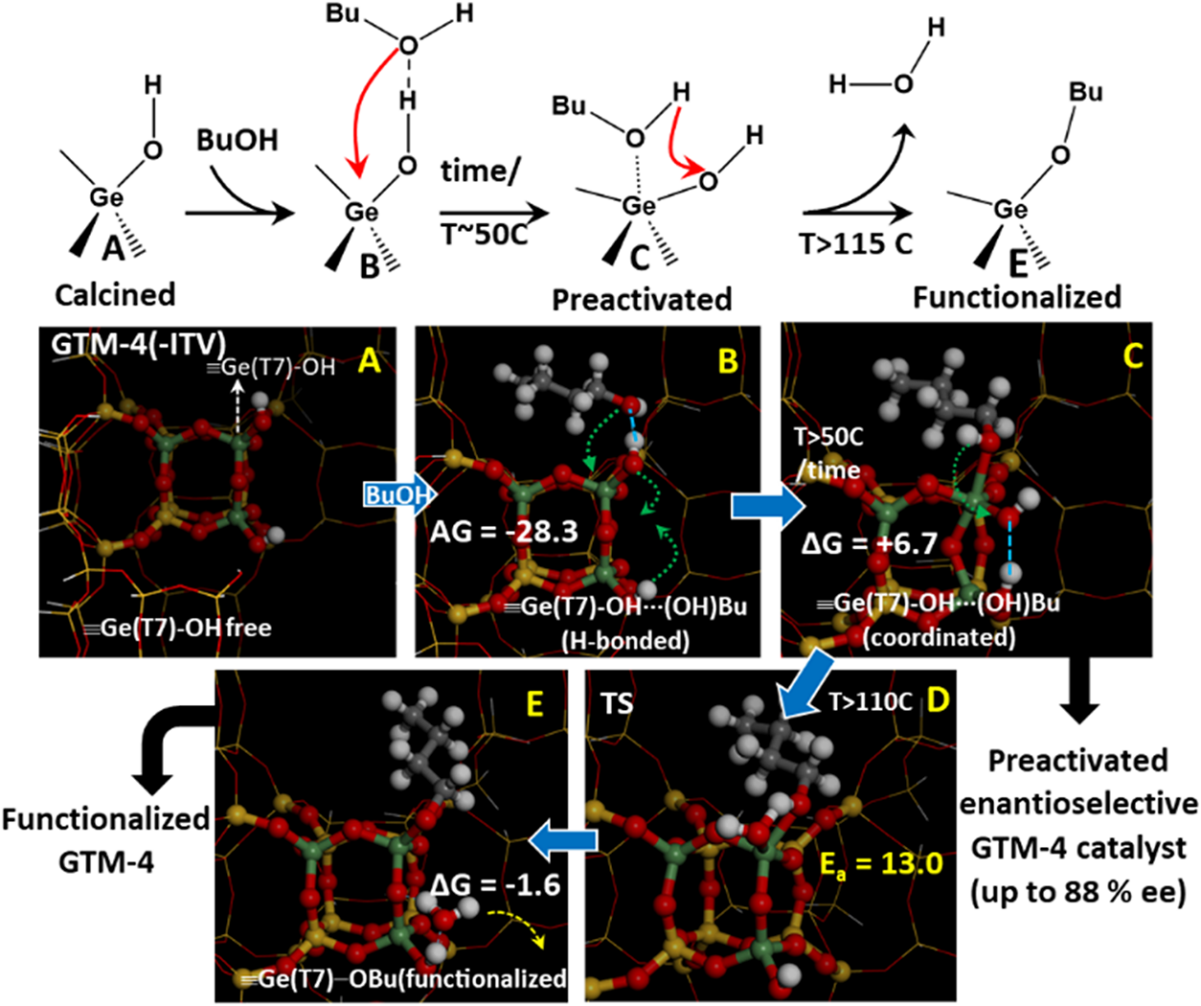
Route for the preactivation of Ge­(T7)­OH sites with butanol and subsequent butoxide-functionalization.

We then wanted to understand how bound butanol molecules evolve toward butoxide species, as observed experimentally. Butanol bound to ≡Ge­(T7)­OH can transfer its proton to one of the three adjacent framework OGe­(T7) atoms in equatorial position (Figure 7C) (the forth is on the opposite axial position, too far as to enable the H-transfer): to Ge(7)–O–Ge(6) (Figure S12 in the Supporting Information, top), to Ge(7)–O–Si(4) (Figure S12, bottom), or to the ≡Ge(7)­OH site (Figure 7C→D). The former two, H-transfer from bound butanol to Ge(7)–O–Ge(6) (Figure S12-top) or to Ge(7)–O–Si(4) (Figure S12-bottom), involve the formation of a Brønsted acid site; both processes require activation barriers around 12–13 kcal/mol, and are endothermic (with free energies at 298 K of +3.8 and +9.5 kcal/mol, respectively), being much less favored the H-transfer to Ge(7)–O–Si(4). These mechanisms to produce Brønsted acid sites should be apparent in the FTIR spectra, but we did not observe such bands, thus suggesting that they should not occur to a large extent upon butanol adsorption. The alternative H-transfer to the interrupted Ge­(T7)­OH site to produce a H_2_O molecule (Figure 7C→D→E) is more favored (free energy of −1.6 kcal/mol), and requiring a similar activation barrier of 13 kcal/mol; in this case, the H_2_O molecule produced is finally released from Ge (Figure 7D), leaving a butoxide species bound to Ge­(T7) that recovers the tetrahedral coordination. This mechanism thus provides a pathway to generate butoxide species after dehydration of Ge­(T7), being more favorable than the H-transfer to framework O’s to form Brønsted acid sites (see Figure S13 in the Supporting Information). Indeed, we found another plausible pathway for the formation of butoxide species by H-transfer to ≡Ge­(T7)–OH sites that involves the assistance of an additional nearby butanol molecule (Figures S14 and S15 in the Supporting Information), with a similar energy profile.

## Discussion

The limited hydrothermal stability of calcined Ge-containing zeolites is a well-known drawback of these materials, especially if high amounts of Ge are present in the framework.^39^ This is associated with the hydrolysis of Ge sites by water molecules due to the Lewis acidity of tetrahedral Ge, which eventually results in a lixiviation of Ge from the zeolite network and the subsequent collapse of the framework.^40^ Such limited stability has traditionally hindered the application of these germanosilicate extra-large-pore materials. Indeed, this framework instability is particularly critical in the -ITV structure due to its very open-framework combined with the high density of terminating OH hydrophilic groups associated with the interrupted positions (32 out of 192 T atoms in the -ITV unit cell), which results in a rapid adsorption of water after elimination of the organics and a subsequent instantaneous framework collapse upon exposure to ambient humidity. In this context, our study shows the interesting effect that the addition of butanol molecules to calcined GTM chiral zeolites has not only on the framework stability but also on the asymmetric catalytic activity. Several *in situ* techniques have enabled us to unravel the role of butanol on the Ge-interrupted sites. Initially, butanol molecules are adsorbed within the -ITV pores through H-bond interactions associated with ≡GeOH interrupted positions. The singular configuration of the ≡Ge–OH sites characteristic of the -ITV framework provokes an enhanced Lewis acidity to such Ge positions due to their inherent higher flexibility. Hence, after the initial adsorption through formation of H-bonds, butanol molecules bind to Ge to form the corresponding Ge···(OH)­Bu complex with an increase of the coordination environment of Ge; this occurs either through a slight increase of the temperature (to 50–70 °C), or probably also after sufficient time. Results on chemical composition (with a close to 1 ratio of butanol/≡Ge–OH) indicate that one butanol molecule binds each ≡GeOH site, thus suggesting the formation of pentacoordinated Ge sites. Indeed, higher than tetrahedral coordination of Ge sites has been recently found in other germanosilicate zeolites (Ge–MFI).^38^ In any case, the formation of such Ge···(OH)­Bu complexes provides a notable resistance of this framework toward hydrolysis, which is particularly important for these highly hydrophilic materials, possibly by hindering the incorporation of water molecules into the pores due to the hydrophobicity introduced with butanol and the lack of free space for water to attack Ge. In consequence, these butanol-loaded GTM materials can then be manipulated without the need for an inert atmosphere. Similarly, we found that smaller alcohols such as methanol also provide the same type of stabilization, yielding a close to 1:1 ratio of methanol/≡GeOH sites, despite its smaller size.

Furthermore, these butanol ligands can be irreversibly anchored to the Ge sites by increasing the temperature beyond 115 °C, which triggers a dehydration reaction that produces irreversibly bound butoxide species. Hence, the same strategy could be used to modify the surface properties of these catalysts, for instance by enhancing the hydrophobicity after anchoring organic groups that would remove the hydroxyl groups of ≡Ge–OH sites, or especially to introduce different types of functionalities within these materials by anchoring species bearing functional groups, which could induce new types of acid, basic, or redox sites, or even to anchor other active species like metallic nanoclusters, as well as to fine-tune the porosity of this framework. Such a strategy of stabilization and subsequent functionalization might also be extended to other germanosilicate interrupted frameworks, which typically contain extra-large pores (like -CLO, -IFT, -IFU, -IRT, -IRY, or -SYT), opening a new way to induce advanced catalytic functionalities in these thrilling open-framework materials.

Interestingly, the adsorption of butanol molecules and the formation of the Ge···(OH)­Bu complexes promote a preactivation of such singular ≡GeOH sites for asymmetric catalytic reactions by increasing their coordination environment, resulting in a new type of Ge sites that boost the enantioselective properties of these catalysts. In the absence of a specific preactivation, the transformation of the initial Ge sites into the highly enantioselective Ge sites by increasing their coordination environment should occur along the course of the reaction (considering that it takes place in the presence of butanol as reactant/solvent), which would explain the increase of the *ee*’s observed in the nonpreactivated samples with conversion/time (Figure 1A-red lines). On the other hand, if such preactivation is performed at 120 °C (instead of at room temperature), where coordinated butanol molecules transform into irreversibly bound butoxide species after dehydration, the catalysts become inactive, suggesting that the enantioselective active sites are given by those ≡GeOH sites coordinated with butanol molecules. Importantly, through this strategy of Ge preactivation by butanol at room temperature, we reached *ee*’s as high as 88% that are unprecedented in zeolite materials; this shows that one TSO enantiomer reacts ∼16 times more than the other, indicating that the formation of these highly coordinated Ge sites strongly enhances the enantio-discrimination ability. Moreover, the enantioselectivities attained with our chiral zeolite materials are now close to those achieved by the archetypic enzymatic catalysts, approaching the potential application of our materials for industrial asymmetric catalysis.

We then wondered about the fundamental chemical reason for such a notable effect of the preactivation treatment on the enantioselective activity of these catalysts. The multitechnique study reported here indicates that the enantioselective active sites for the S_N_2 ring-opening of TSO are pentacoordinated ≡GeOH interrupted sites with one butanol bound as ligand under these preactivation conditions, in contrast to the mechanism originally proposed that involved an initial coordination of TSO on tetrahedral Ge.^24^ We then envisage three potential routes for the subsequent ring-opening reaction of TSO with butanol. The first route would involve a further increase of the Ge coordination to an octahedral environment to bind and activate a TSO molecule. However, hexacoordination of Ge in this framework seems unlikely, as evidenced by the fact that there seems to be a 1:1 ratio of ≡GeOH:BuOH, even at high partial pressures of BuOH, which discards the coordination of more than one BuOH molecule per GeOH site, probably because of a strong steric hindrance. Moreover, should a further increase of the coordination environment be possible, this would trigger the hydrolysis mechanism by binding water molecules, which we have shown is not the case. Besides, DFT results show a lower adsorption energy for coordinating an additional butanol ligand (see Figure S14 in the Supporting Information). Thus, direct coordination of TSO on ≡GeOH···(OH)­Bu to give Ge octahedral sites seems to be ruled out. A second route would involve a release of the butanol ligand to revert to tetrahedral Ge, which would then be able to bind TSO molecules, but this route would be in disagreement with the preactivation results reported here. Furthermore, we have observed that in the absence of butanol (where ≡GeOH sites are in a tetrahedral environment), TSO is rapidly transformed within our catalysts in diphenylacetaldehyde through the *Meinwald* rearrangement reaction. This suggests that direct coordination of TSO to tetrahedral Ge results in the formation of a carbocation-like species through the S_N_1 route (as has been recently reported for propylene oxide on Ge–MFI),^41^ which is favored in the absence of nucleophiles and also under certain conditions like high temperature, presence of solvents other than the alcohol, or high TSO concentration.^21^ Thus, we propose pentacoordinated ≡GeOH···(OH)­Bu sites as the real active site for the enantioselective S_N_2 ring-opening reaction to yield the *unlike* products, where GeOH···(OH)­Bu sites would attack free (unbound) TSO molecules confined within the -ITV pores.

The crucial question that remains unanswered is to understand how these pentacoordinated ≡GeOH···(OH)­Bu sites trigger the highly enantioselective S_N_2 ring-opening of TSO. Throughout our studies, we have noticed that the mechanism of action of our zeolite chiral catalyst seems to be related to that of enzymes, in particular considering the confined nanospace where the catalytic reaction takes place on a singular active site surrounded by a confined asymmetric pocket/cavity given by the chirality of the surrounding amino acid residues (in the case of enzymes) or framework walls (in the case of our zeolite material), and also in terms of the potential modification of solvents or other cofactors that can alter the structure of the active site and promote their enantioselective properties. For this reason, we looked for inspiration in nature by analyzing the chiral molecular mechanism of highly enantioselective enzymes on related reactions, in particular for the ring-opening of *trans*-stilbene or *cis*-stilbene oxides with water to form the corresponding diols, which is catalyzed by epoxide hydrolases through a well-established mechanism.^42–45^ In this asymmetric catalytic reaction, the ring-opening of stilbene oxide takes place within the confined chiral space provided by the particular positioning and orientation of several amino acid residues with catalytic action that generate an asymmetric environment (the so-called *catalytic triad*).^45^ Specifically, the main role of the chiral biological catalyst is to promote a particular orientation of the bulky epoxide reactant, determined by the development of H-bonds with surrounding hydroxyl groups of tyrosine or aspartic acid side-chains, while keeping a close contact with the nucleophile that will boost the S_N_2 reaction.^42^ Such a particular orientation of the epoxide is favored for one specific enantiomer of TSO, which will determine their relative trend to react and consequently the resulting catalytic enantioselectivity. In our zeolite system, the reaction mechanism takes place from the activated butanol molecule linked to ≡Ge­(OH) sites that attack free TSO reactant molecules, as previously discussed. This scenario resembles the chiral mode of action of such epoxide hydrolases, where the epoxide locates in a particular orientation determined by the development of H-bonds with surrounding hydroxyl-containing residues. Given the chirality of the -ITV framework walls and the abundant occurrence of framework hydroxyl groups, a similar mechanism could be envisaged for our inorganic chiral catalyst.

In an attempt to address this issue, we performed a combined force field/DFT study to understand how catalytic enantioselectivity could emerge within the -ITV framework under these preactivation conditions. Following the computational work applied to these enzymatic systems,^42^ we define the emergence of enantioselectivity as the potential to promote simultaneously (i) an activation of the TSO oxirane ring by formation of an H-bond with a surrounding hydroxyl group, that would partially polarize the ring to promote its reactivity and stabilize the resulting ring-opening product, while (ii) locating close to the activated nucleophilic butanol molecule and in an adequate orientation to trigger the attack to the C atom of the oxirane ring from the opposite side.

In our case, similar to the case of hydrolase enzymes, the abundance of ≡GeOH···(OH)­Bu (T7 and T8 positions) associated with *d4r* units involves that they locate close to each other, with shortest framework O­(H)···O­(H) distances around 7.5 Å (see Figure 8). Our calculations showed that bulky TSO molecules tend to site aligned with the -ITV pores just in-between two ≡GeOH···(OH)­Bu sites associated with nearby *d4r* units (see Figure 8-left for *RR*-TSO and right for *SS*-TSO), stabilized by interactions with the framework walls. Interestingly, a crucial difference between both enantiomers turns up. In the case of *RR*-TSO, the most stable orientation found in our calculations involves the development of a strong H-bond with a framework hydroxyl group from the nearby ≡GeOH···(OH)­Bu site (Figure 8-left, dashed blue line), while keeping the oxirane ring in an adequate orientation to promote the nucleophilic attack of preactivated BuOH (which could even be deprotonated by H-transfer to nearby framework O atoms, as previously discussed) from the opposite side of the ring (red dashed arrow in Figure 8-left), thus satisfying both conditions for the S_N_2 reaction to occur. In contrast, in the most stable orientation found for *SS*-TSO, such a stabilizing H-bond interaction is not established (Figure 8-right), and indeed the orientation of the oxirane ring (with the O of the oxirane ring facing preactivated butanol) would prevent the nucleophilic attack, hindering the S_N_2 ring-opening reaction for this enantiomer. MD simulations confirmed that such an H-bond is exclusively preserved for the *RR*-TSO enantiomer (Figure S16 in the Supporting Information). Hence, the different orientation of both TSO enantiomers triggered by the confined chiral space of the preactivated -ITV framework and the development of specific H-bond interactions with nearby hydroxyl framework groups would result in the emergence of enantioselectivity in our chiral zeolite catalyst, in a very similar fashion to that occurring in epoxide hydrolase enzymes. Of course, the specific orientations proposed here represent just one example, and given the complexity of the preactivated -ITV system, we do not mean that these will be the actual and unique orientations occurring, but still they nicely illustrate how enantioselectivity can emerge from our preactivated chiral zeolite catalysts.

**Figure 8:**
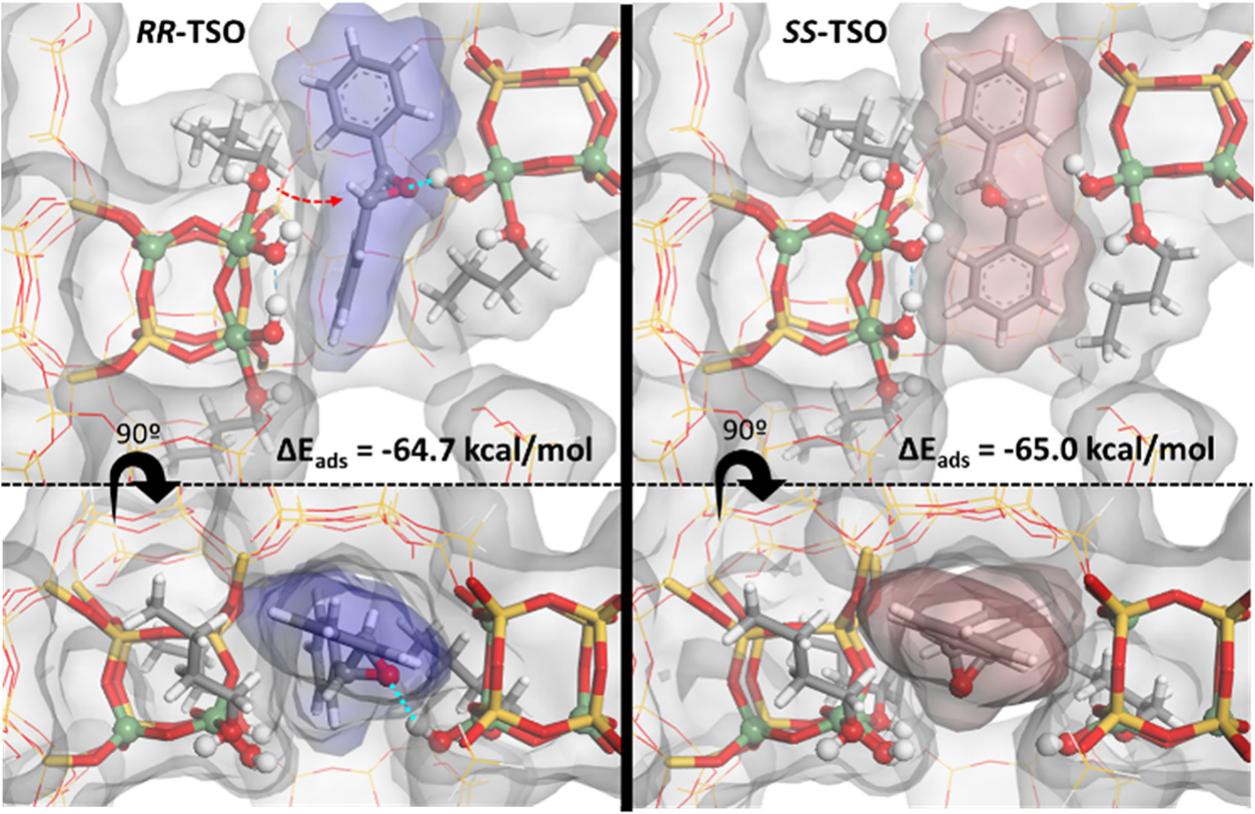
Two perpendicular views (top and bottom) of the DFT-calculated most stable orientation of *RR*-TSO (left, blue shadow) and *SS*-TSO (right, orange shadow) in butanol-preactivated *P*4_1_32 −ITV framework (butanol in T8 coordinated from below); TSO···H­(O)Ge H-bonds are displayed as dashed blue lines; *d4r* nearby units are highlighted with atoms displayed as sticks (Si and O) and balls (Ge). Color code: Si (yellow); Ge (green); O (red); C (gray); H (white).

## Conclusions

A pretreatment of GTM asymmetric catalysts based on the -ITV framework carried out by submerging the calcined samples in 1-butanol has proven to be a very efficient methodology to, on the one hand, stabilize the materials against attack of water and, on the other and most importantly, promote a preactivation of the chiral Ge active sites that boosts their enantioselective catalytic activity, allowing reaching *ee* values close to 90%. A combination of *in situ* FTIR, mass spectrometry, and synchrotron-based EXAFS/XANES techniques revealed that upon adsorption of butanol by calcined GTM-4 catalysts, butanol molecules first interact predominantly with ≡GeOH interrupted positions through H-bond interactions, and after some time and/or a slight increase of the temperature (to 50–70 °C), these butanol molecules start to coordinate to the Ge atom of the ≡GeOH sites, prompting an increase of its coordination number and a partial distortion of the Ge environment that notably enhances the enantioselective properties. Indeed, the actual enantioselective catalytic active sites are these highly coordinated ≡GeOH sites that are produced *in situ* upon butanol adsorption, while uncoordinated Ge sites seem to be either nonenantioselective or inactive for the S_N_2 asymmetric ring-opening reaction. Interestingly, further heating of these coordinated butanol molecules (beyond 100 °C) triggers a dehydration reaction with the interrupted hydroxyl groups that releases water and involves a factual transformation of hydroxyl in butoxide species bound to Ge, recovering the tetrahedral Ge coordination. Hence, these simple treatments with 1-butanol involve not only a stabilization of the catalyst against humidity and an exceptional enhancement of the asymmetric catalytic activity but also a route to easily functionalize these materials with different groups allowing modification of the surface properties, both in terms of pore size and hydrophobic properties, and also to incorporate new advanced catalytic functionalities in these chiral materials. All of these interesting properties of the -ITV framework seem to be connected to the singularities of the interrupted positions occupied by Ge in these materials, promoting an emergence of chirality in a fashion similar to that occurring in related enzymes.

## Supplementary Material



## Notes

Reaction schemes, further catalytic data, physicochemical characterization, images of DFT-geometry-optimized structures, energy profiles, and MD simulation results are associated with this work (PDF)
