# Supplementary material for: Enzyme-like Enantioselectivity in GTM Chiral Zeolite Catalysts upon Preactivation of Ge Sites
Source: J Am Chem Soc. 2025 Oct 8;147(42):38585–98. doi: 10.1021/jacs.5c12567 (PMC12550838; doi:10.1021/jacs.5c12567)
Supplement: Supplementary file 1 [file ja5c12567_si_001.pdf]

Supporting Information

Enzyme-like enantioselectivity in GTM chiral zeolite catalysts upon preactivation of Ge sites

*Ramón de la Serna,[1] Jaime Jurado-Sánchez,[1] Carlos Márquez-Álvarez,[1] M. Asunción Molina,[2,3] Lucy Costley-Wood,[2,3] Andrew M. Beale,[2,3] Diego Gianolio,[4] Joaquín Pérez-Pariente,[1] and Luis Gómez-Hortigüela[1]**

[1] Instituto de Catálisis y Petroleoquímica, Consejo Superior de Investigaciones Científicas (ICP-CSIC), c/ Marie Curie 2, 28049 Madrid, Spain. Email: lhortiguela@icp.csic.es

[2] Department of Chemistry, University College London, 20 Gordon Street, WC1H 0AJ, UK

[3] Research Complex at Harwell, Rutherford Appleton Laboratories, Harwell Science and Innovation Campus, Harwell, Didcot OX11 0FA, UK

[4] Diamond Light Source, Harwell Science and Innovation Campus, Didcot OX11 0DE, UK

Table of Contents

Scheme S1. Ring-opening reaction of chiral (1*R*,2*R*) (top) or (1*S*,2*S*) (bottom) *trans*-stilbene oxide with 1-butanol, yielding major chiral *unlike*-products (*R,S*+*S,R*) or minor *like*-products (*R,R*+*S,S*); species in columns are enantiomers.

Figure S1. Asymmetric catalytic activity (reported as enantiomeric excess, *ee*) of calcined GTM-3 (prepared from (1*S*,2*S*)-N-ethyl-N-methylephedrinium hydroxide, *SS*-EMPS) as-made (red lines) and after preactivation (blue lines) catalysts for the *trans*-stilbene oxide ring-opening with 1-butanol (at room temperature).

Figure S2. Asymmetric catalytic activity (reported as enantiomeric excess) of calcined GTM-4 (prepared from (1*S*,2*S*)-N-methyl-N-(2-methylbenzyl)pseudoephedrinium hydroxide, *SS*-OMBMPS) as-made (red lines) and after preactivation (blue lines) catalysts for the *trans*-stilbene oxide ring-opening with 1-butanol (at room temperature).

Figure S3. Selectivity for the *unlike* (top) and *like* (bottom) products of GTM-4 (prepared from (1*R*,2*S*)-N-methyl-N-(2-methylbenzyl)ephedrinium hydroxide, *RS*-OMBMEP) as-made (red lines) and after preactivation (blue lines) catalysts for the *trans*-stilbene oxide ring-opening with 1-butanol (at room temperature).

Figure S4. Opposite asymmetric catalytic activity (reported as enantiomeric excess of reactants, top, and main *unlike* products, bottom) of preactivated antipode GTM-4 catalysts prepared from (1*R*,2*S*)-N-methyl-N-(2-methylbenzyl)ephedrinium hydroxide (*RS*-OMBMPM, red lines) or (1*S*,2*R*)-N-methyl-N-(2-methylbenzyl)ephedrinium hydroxide (*SR*-OMBMPM, blue lines).

Figure S5. Comparison of the asymmetric catalytic activity (reported as enantiomeric excess) of the different GTM catalysts after preactivation with 1-butanol (at room temperature).

Figure S6. Thermogravimetric analysis of GTM-4 after submersion in 1-butanol at 50 or 115 °C.

Figure S7. XRD patterns of GTM-4(*RS*-OMBMEP) after preactivation in butanol at 115 °C. From bottom to top: as-made, calcined (performed under inert atmosphere), calcined (immediately after exposure to ambient air), and after 24, 96 and 168 hours under exposure to air.

Figure S8. Relative intensity of bands at *ca*. 2960 and 2940 cm^{-1} for the whole series of FTIR spectra recorded in the TPD-FTIR analysis of sample GTM-4(*RS*-OMBMEP) with 1-butanol adsorbed at room temperature (selected spectra shown in Figure 4 of the main manuscript).

Figure S9. ^{13}C CP MAS NMR of GTM-4(*RS*-OMBMEP) catalyst after preactivation in butanol at 50 (blue line) or 115 °C (red line).

Figure S10. Additional normalized XANES spectra at the Ge K-edge for GTM-4 throughout the in-situ experiment. Left: during heating of the sample from room temperature up to 90 °C (butanol coordination to Ge); right: during heating from 90 up to 200 °C (dehydration and butoxide formation). In both cases, an inset has been added showing the region around 11122 eV magnified to highlight the evolution of the post-edge feature.

Figure S11. Geometry-optimized structure of 1-butanol molecules interacting with the framework ≡Ge-OH (T7) sites through H-bonds; two different orientations are shown. Adsorption free energies (AG) are given in kcal/mol.

Figure S12. Evolution of bound butanol by H-transfer to framework O adjacent to Ge(7) and Ge(6) (top) or to framework O adjacent to Ge(7) and Si(4) (bottom); activation and free energies given in kcal/mol (calculated at 298 K).

Figure S13. Energy profile for the evolution of bound butanol by H-transfer to framework O adjacent to Ge(7) and Ge(6) (blue), to framework O adjacent to Ge(7) and Si(4) (orange) or to Ge(7)OH (grey) (without additional butanol).

Figure S14. Relative free energies (calculated at 298 K) for the addition of a second butanol molecule.

Figure S15. Alternative potential route for the formation of butoxide species after H-transfer to ≡Ge(T7)-OH with the assistance of a second butanol molecule.

Figure S16. Evolution (left) and distribution (right) of the H-bond O(TSO···H(OGe)≡ distance along MD simulations for RR-TSO (blue lines) and SS-TSO (orange lines).

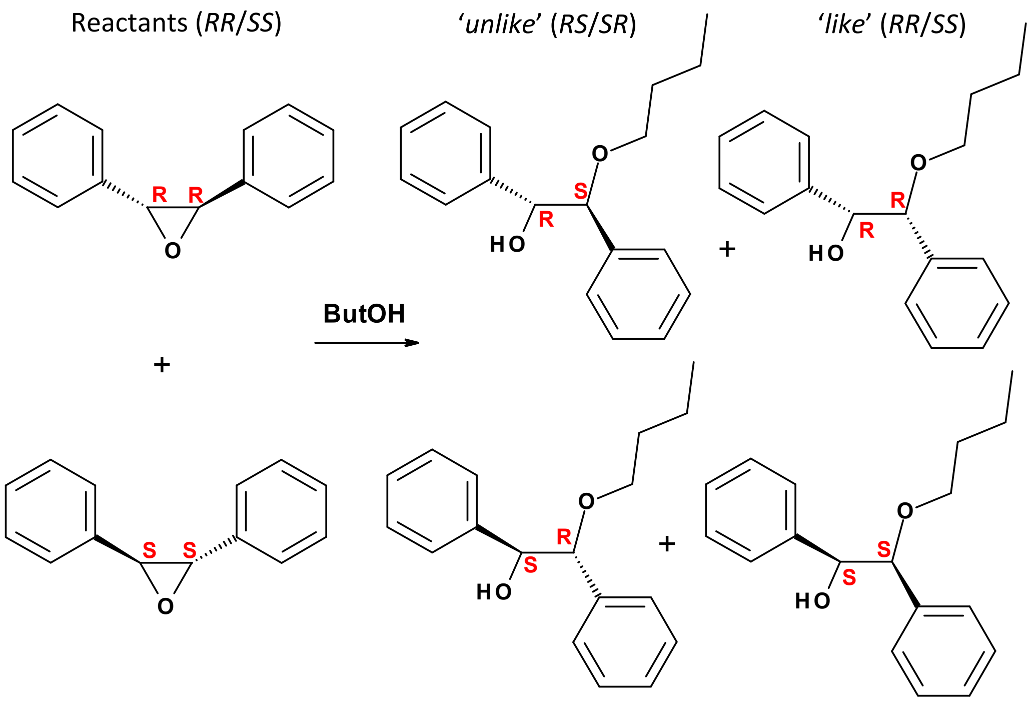

Scheme S1. Ring-opening reaction of chiral (1*R*,2*R*) (top) or (1*S*,2*S*) (bottom) *trans*-stilbene oxide with 1-butanol, yielding major chiral *unlike*-products (*R*,*S*+*S*,*R*) or minor *like*-products (*R*,*R*+*S*,*S*); species in columns are enantiomers.

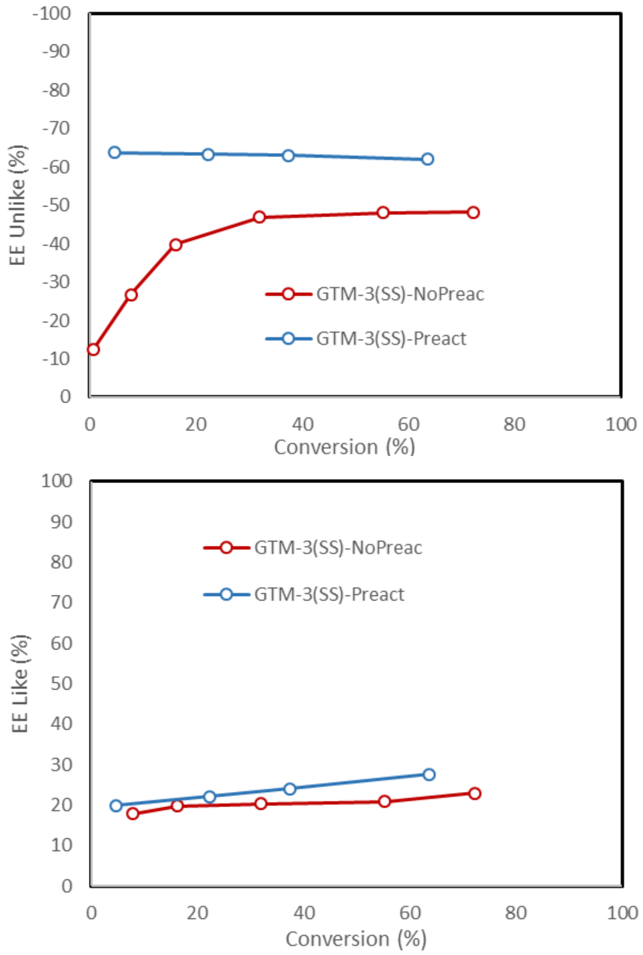

Figure S1. Asymmetric catalytic activity (reported as enantiomeric excess, ee) of calcined GTM-3 (prepared from (1*S*,2*S*)-N-ethyl-N-methylephedrinium hydroxide, *SS*-EMPS) as-made (red lines) and after preactivation (blue lines) catalysts for the *trans*-stilbene oxide ring-opening with 1-butanol (at room temperature).

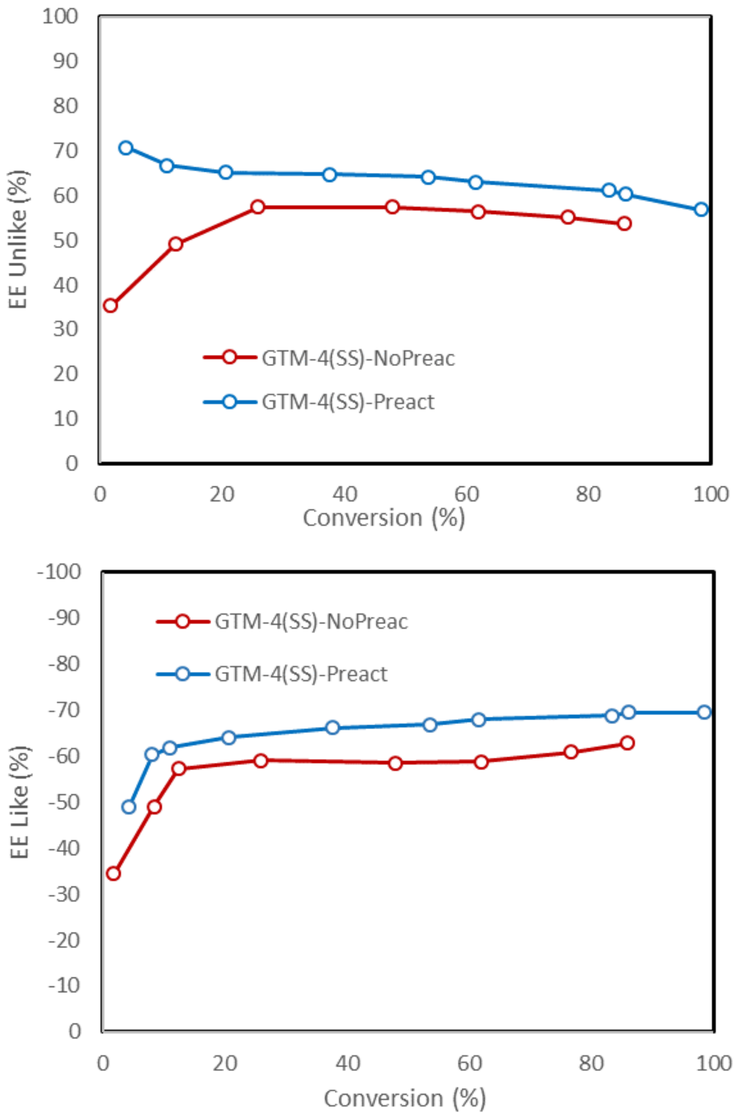

Figure S2. Asymmetric catalytic activity (reported as enantiomeric excess) of calcined GTM-4 (prepared from (1*S*,2*S*)-N-methyl-N-(2-methylbenzyl)pseudoephedrinium hydroxide, *SS*-OMBMPS) as-made (red lines) and after preactivation (blue lines) catalysts for the *trans*-stilbene oxide ring-opening with 1-butanol (at room temperature).

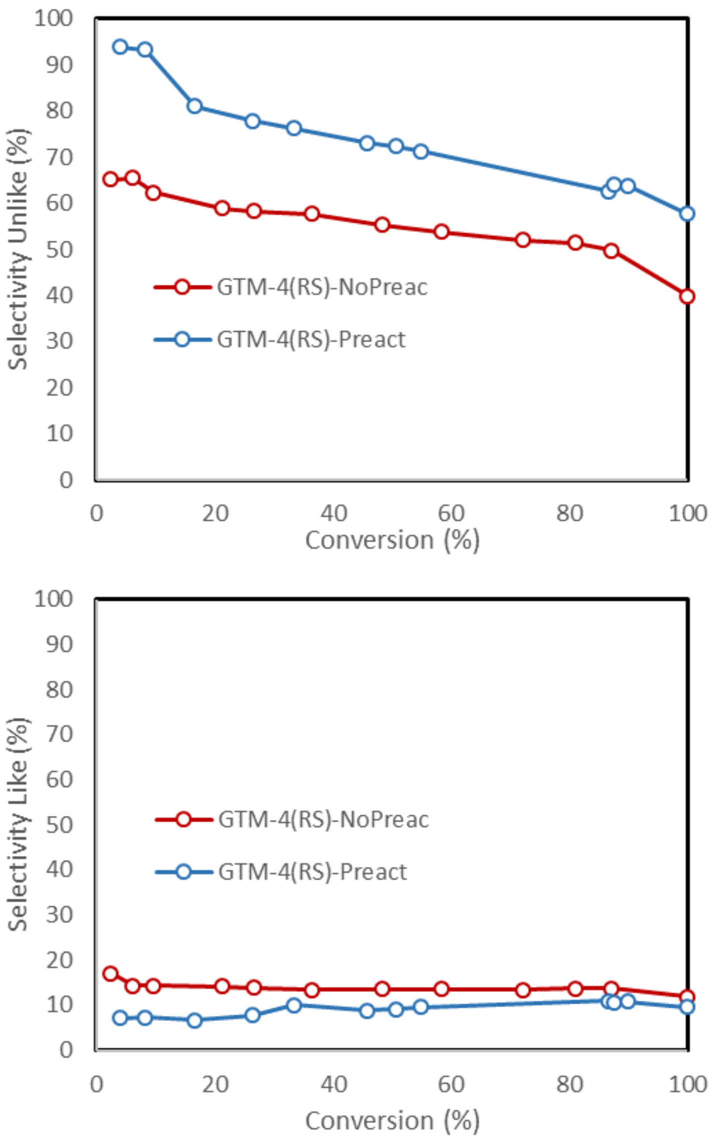

Figure S3. Selectivity for the *unlike* (top) and *like* (bottom) products of GTM-4 (prepared from (1*R*,2*S*)-N-methyl-N-(2-methylbenzyl)ephedrinium hydroxide, *RS*-OMBMEP) as-made (red lines) and after preactivation (blue lines) catalysts for the *trans*-stilbene oxide ring-opening with 1-butanol (at room temperature).

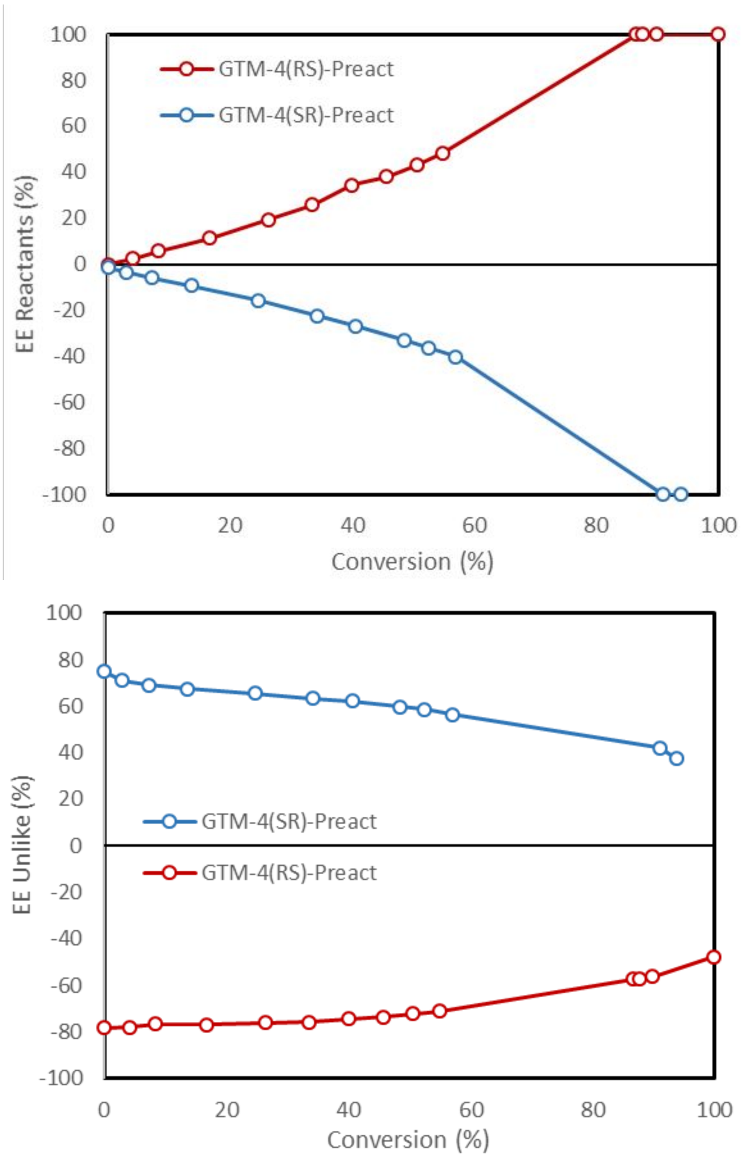

Figure S4. Opposite asymmetric catalytic activity (reported as enantiomeric excess of reactants, top, and main *unlike* products, bottom) of preactivated antipode GTM-4 catalysts prepared from (1*R*,2*S*)-N-methyl-N-(2-methylbenzyl)ephedrinium hydroxide (*RS*-OMBMPM, red lines) or (1*S*,2*R*)-N-methyl-N-(2-methylbenzyl)ephedrinium hydroxide (*SR*-OMBMPM, blue lines).

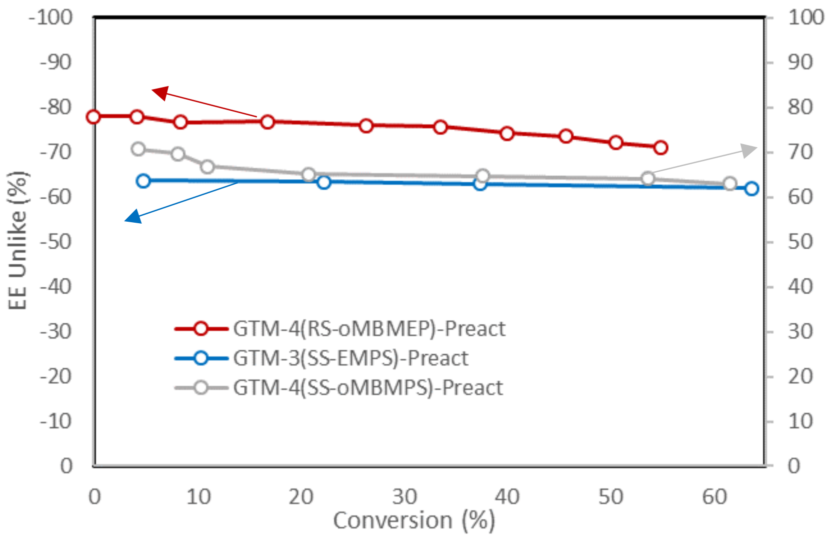

Figure S5. Comparison of the asymmetric catalytic activity (reported as enantiomeric excess) of the different GTM catalysts after preactivation with 1-butanol (at room temperature).

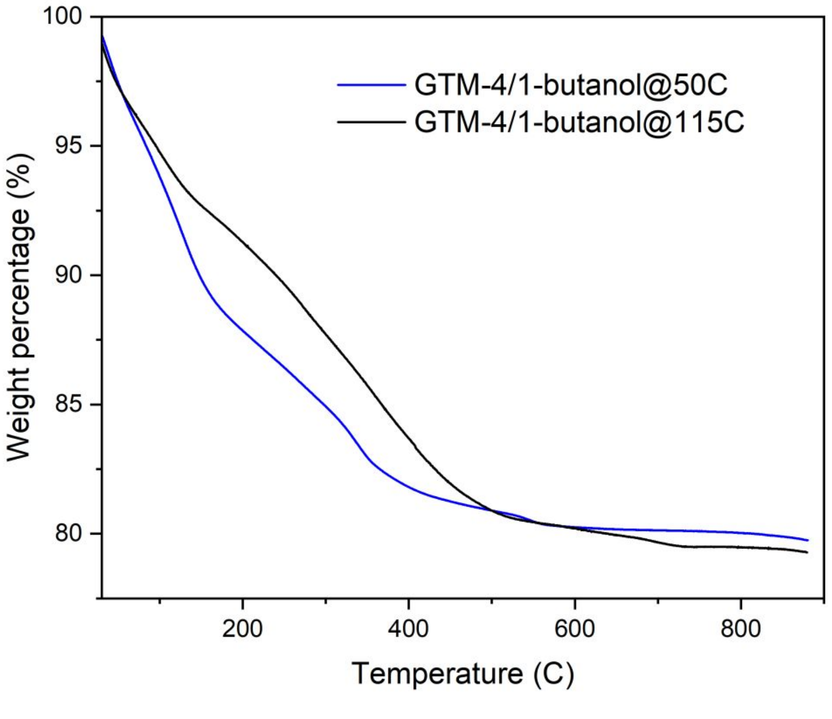

Figure S6. Thermogravimetric analysis of GTM-4 after submersion in 1-butanol at 50 or 115 °C.

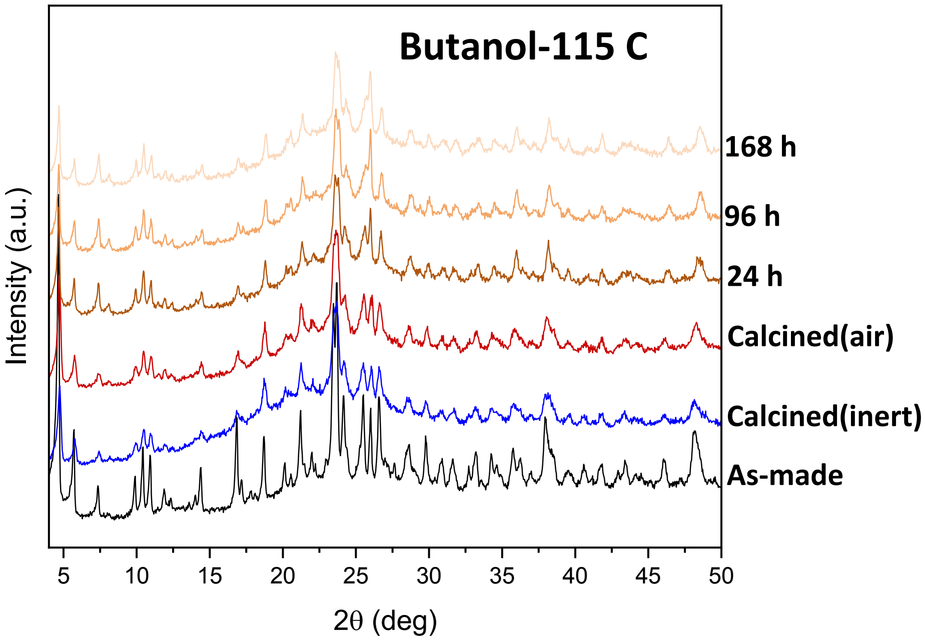

Figure S7. XRD patterns of GTM-4(*RS*-OMBMEP) after preactivation in butanol at 115 °C. From bottom to top: as-made, calcined (performed under inert atmosphere), calcined (immediately after exposure to ambient air), and after 24, 96 and 168 hours under exposure to air.

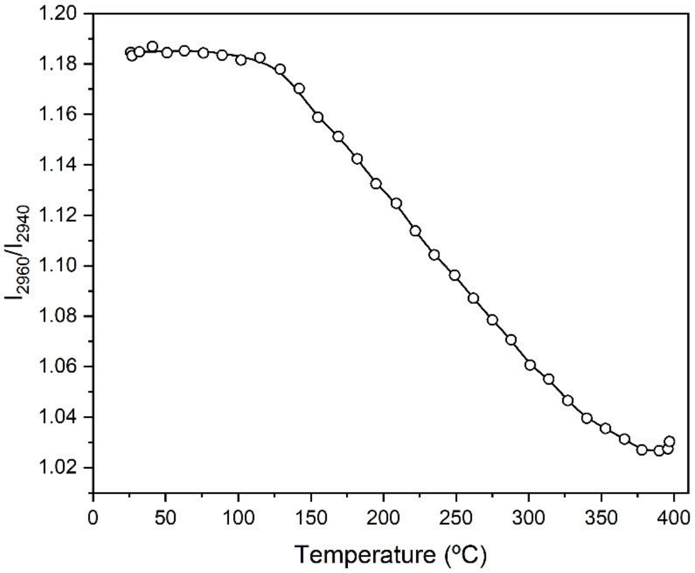

Figure S8. Relative intensity of bands at *ca*. 2960 and 2940 cm^{-1} for the whole series of FTIR spectra recorded in the TPD-FTIR analysis of sample GTM-4(*RS*-OMBMEP) with 1-butanol adsorbed at room temperature (selected spectra shown in Figure 4 of the main manuscript).

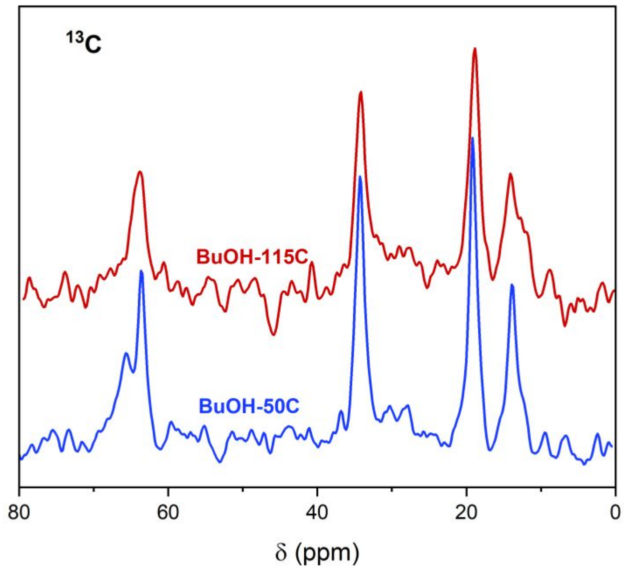

Figure S9. ^{13}C CP MAS NMR of GTM-4(*RS*-OMBMEP) catalyst after preactivation in butanol at 50 (blue line) or 115 °C (red line).

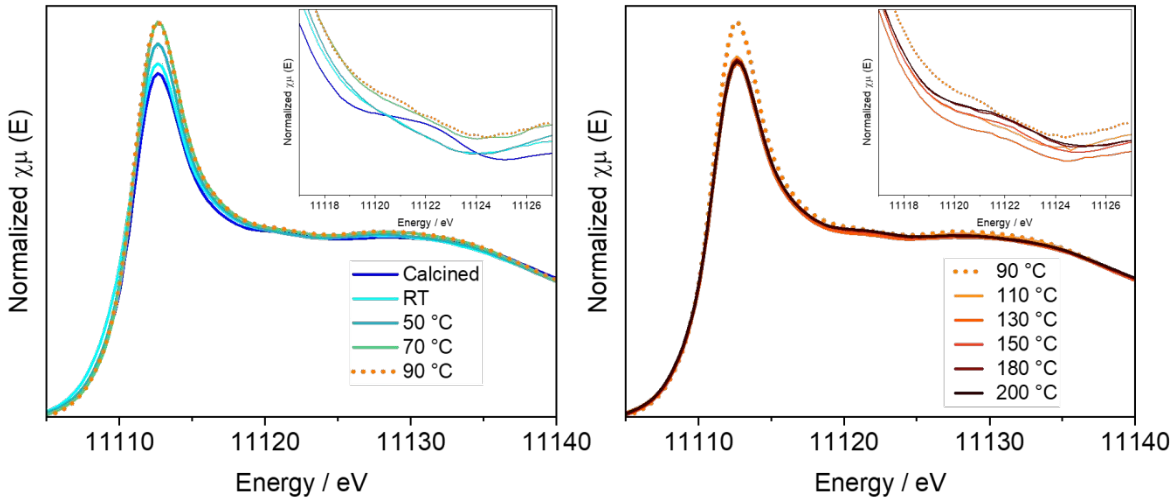

Figure S10. Additional normalized XANES spectra at the Ge K-edge for GTM-4 throughout the in-situ experiment. Left: during heating of the sample from room temperature up to 90 °C (butanol coordination to Ge); right: during heating from 90 up to 200 °C (dehydration and butoxide formation). In both cases, an inset has been added showing the region around 11122 eV magnified to highlight the evolution of the post-edge feature

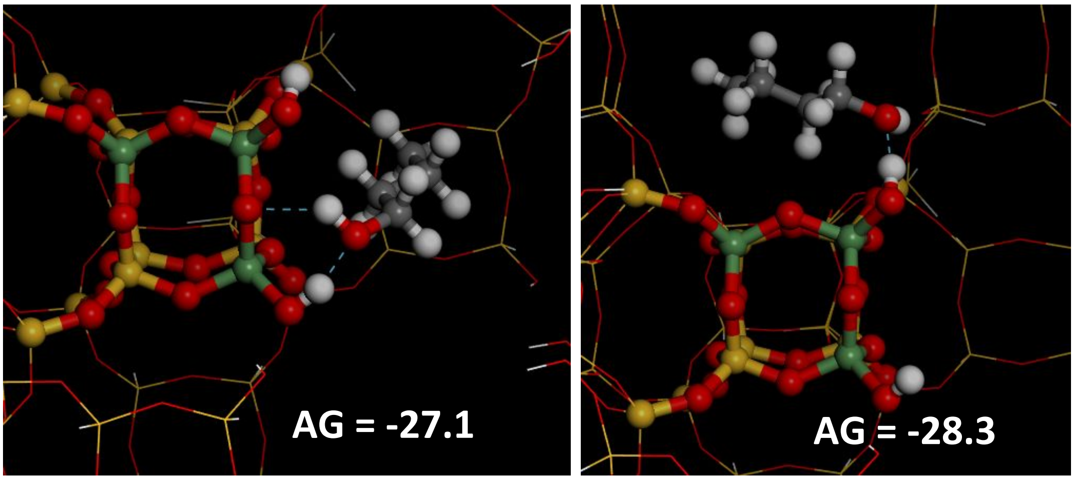

Figure S11. Geometry-optimized structure of 1-butanol molecules interacting with the framework ≡Ge-OH (T7) sites through H-bonds; two different orientations are shown. Adsorption free energies (AG) are given in kcal/mol.

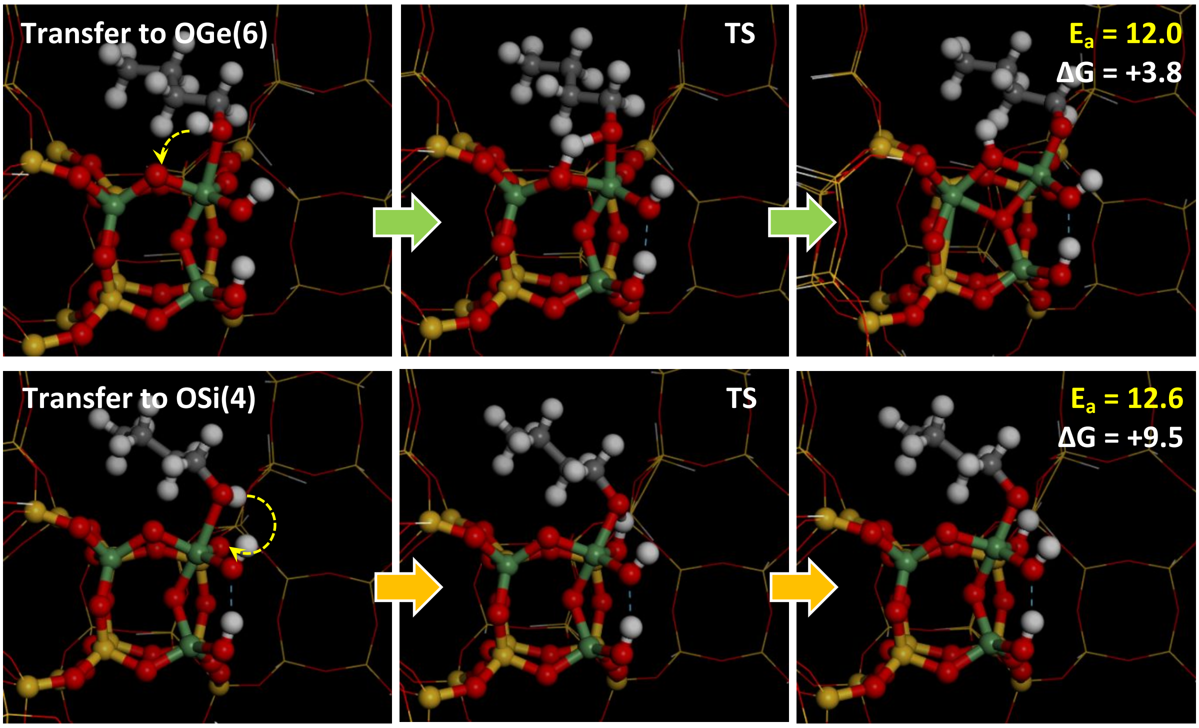

Figure S12. Evolution of bound butanol by H-transfer to framework O adjacent to Ge(7) and Ge(6) (top) or to framework O adjacent to Ge(7) and Si(4) (bottom); activation and free energies given in kcal/mol (calculated at 298 K).

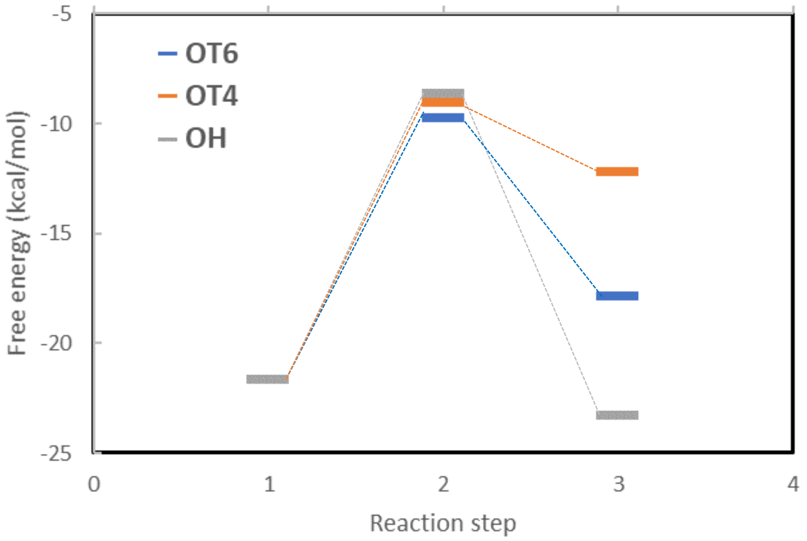

Figure S13. Energy profile for the evolution of bound butanol by H-transfer to framework O adjacent to Ge(7) and Ge(6) (blue), to framework O adjacent to Ge(7) and Si(4) (orange) or to Ge(7)OH (grey) (without additional butanol).

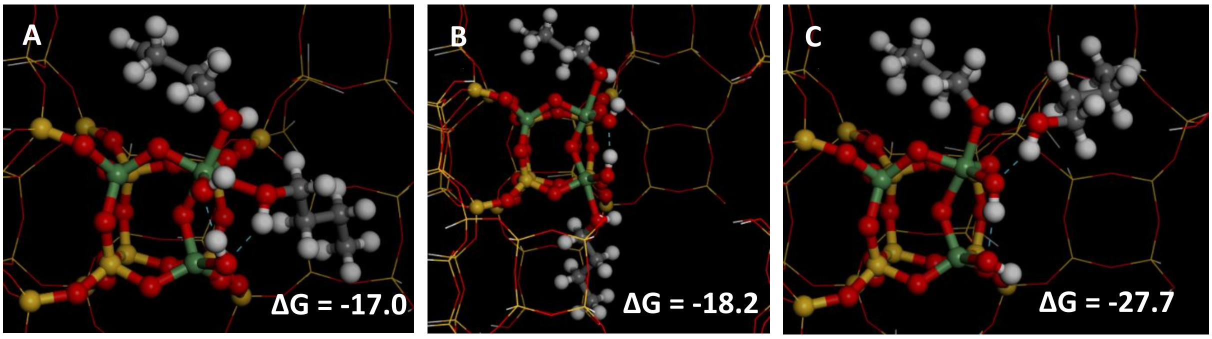

Figure S14. Relative free energies (calculated at 298 K) for the addition of a second butanol molecule.

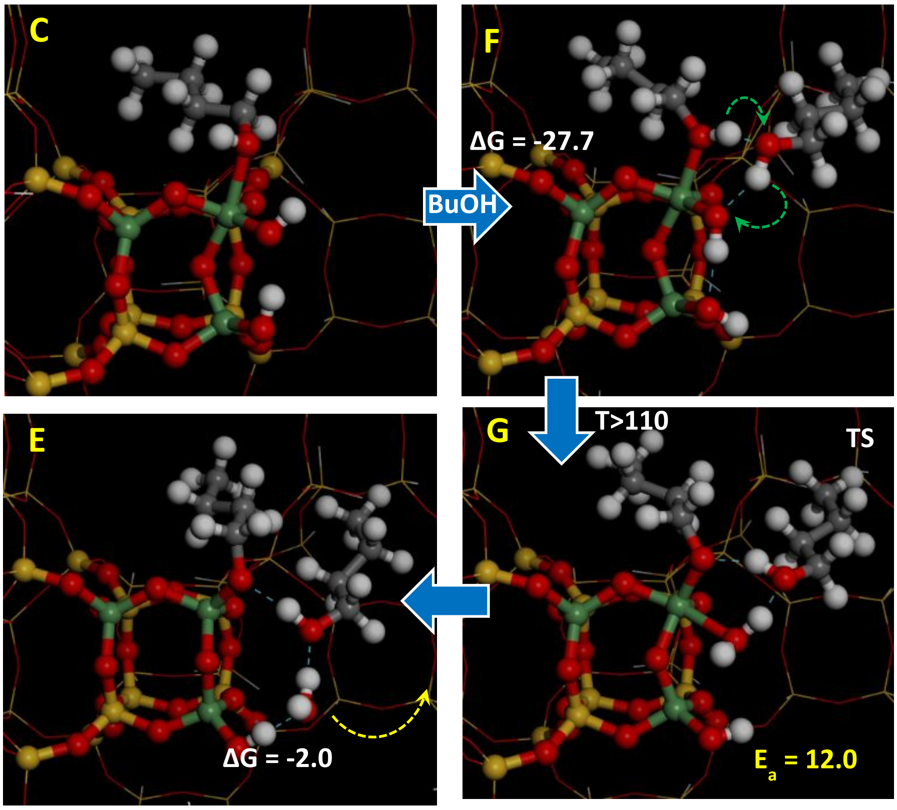

Figure S15. Alternative potential route for the formation of butoxide species after H-transfer to ≡Ge(T7)-OH with the assistance of a second butanol molecule.

Adsorption of a second butanol molecule (Figure S14) can take place via binding to the same Ge that would increase its coordination environment to 6 (octahedral) (A), to the adjacent ≡Ge(T7)-OH site below (B), or alternatively could develop a double H-bond interaction with the first bound butanol molecule and the ≡Ge(T7)-OH site (C), resulting in adsorption free energies of -17.0, -18.2 and -27.7 kcal/mol, respectively. This double-butanol H-bonded host-guest complex displays the appropriate configuration to promote an H-transfer through such H-bonds to the ≡Ge(T7)-OH site to produce a H_2O molecule assisted by the presence of butanol (Figure S15), with a final release of the H_2O molecule. This process displays a similar energy profile to that with only one butanol molecule, providing an alternative route for the formation of butoxide species when large amounts of butanol are available in the pores.

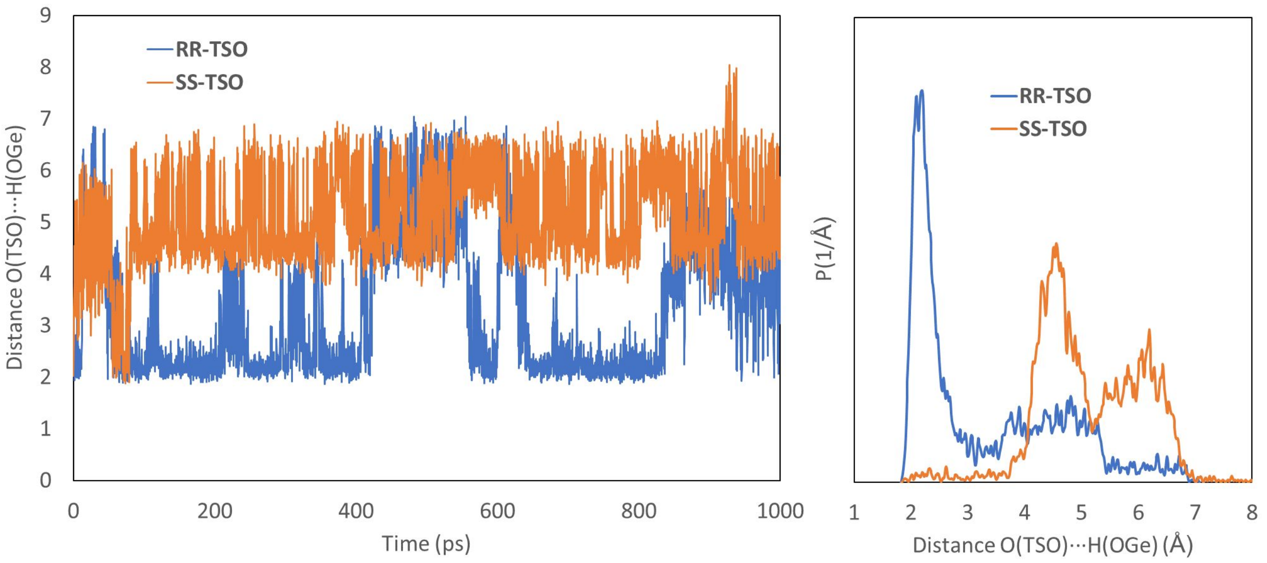

Figure S16. Evolution (left) and distribution (right) of the H-bond O(TSO···H(OGe)≡ distance along MD simulations for RR-TSO (blue lines) and SS-TSO (orange lines).

Dreiding MD simulations (NVT ensemble at 273 K) showed that the H-bond interaction was preserved for *RR*-TSO (blue line, O(TSO)···H(OGe) distance around 2 Å) for most of the simulation time (1 ns), whilst *SS*-TSO (orange line) did not allow for such H-bond formation and disappeared right at the beginning of the simulation (O(TSO)···H(OGe) distance above 4 Å).
